# Molluscan Shells, Spicules, and Gladii Are Evolutionarily Deeply Conserved

**DOI:** 10.1002/jez.b.23294

**Published:** 2025-04-09

**Authors:** Cristian Camillo Barrera Grijalba, Sonia Victoria Rodríguez Monje, Gabriela Ariza Aranguren, Kathrin Lunzer, Maik Scherholz, Emanuel Redl, Tim Wollesen

**Affiliations:** ^1^ Faculty of Life Sciences, Department of Evolutionary Biology University of Vienna Vienna Austria

**Keywords:** biomineralization, gene expression, Hox gene, lophotrochozoa, ontogeny, spiralia, transcription factor

## Abstract

Shells, spicules, and chaetae are diverse among extant and extinct spiralians such as mollusks, annelids, or brachiopods. These hard parts serve different functions, but their formation process and evolutionary interrelationships are still contentious. We investigated the expression of evolutionarily conserved transcription factor encoding genes as well as the structural genes *chitin synthase* and *ferritin* in cells giving rise to shells and spicules of aculiferans, i.e. the polyplacophoran *Acanthochitona fascicularis* and the neomeniomorph *Wirenia argentea*, as well as the conchiferan cephalopod *Xipholeptos notoides* and the scaphopod *Antalis entalis*. Polyplacophorans and neomeniomorphs express *hox1* (only neomeniomorphs), *goosecoid*, *grainyhead*, and *chitin‐synthase* in their spicules. *Grainyhead*, *notch*, *delta*, and *zic* are expressed in the polyplacophoran shell fields and spicule‐bearing cells. In conchiferans, *hox1* (scaphopods and cephalopods), *goosecoid*, and *grainyhead* (scaphopods) are expressed in the shell fields. *Ferritin*, is a gene that has been shown to be expressed in the gastropod shell field; however, it is not expressed in the shell fields or by the spicule‐bearing cells of the studied species. Our study shows that all candidate genes are expressed in epithelia that give rise to spicules and shells, revealing a close relationship between spicule‐bearing cells and shell fields. In contrast, *ferritin* expression in the shell field appears to be a gastropod innovation. Building on previous research involving brachiopod and annelid chaetal sacs, our results suggest that spicules may have predated molluscan shells and may be homologous to brachiopod and annelid chaetae. If this were true, then conchiferan mollusks would have secondarily lost spicules.

## Graphical abstract

Evolutionarily conserved transcription factor encoding genes and other genes are expressed in the epithelia that give rise to spicules (green) and/or shells (blue) in developmental stages of diverse mollusks, including polyplacophorans, aplacophorans, scaphopods, and cephalopods. Building on previous research on brachiopod and annelid chaetal sacs (green), our findings suggest that spicules may have predated molluscan shells and may be homologous to brachiopod and annelid chaetae. If this were true, then conchiferan mollusks would have secondarily lost spicules.

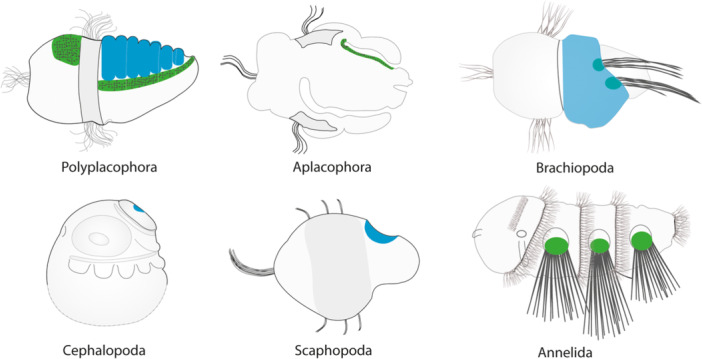

## 1 Introduction

**Figure 1:**
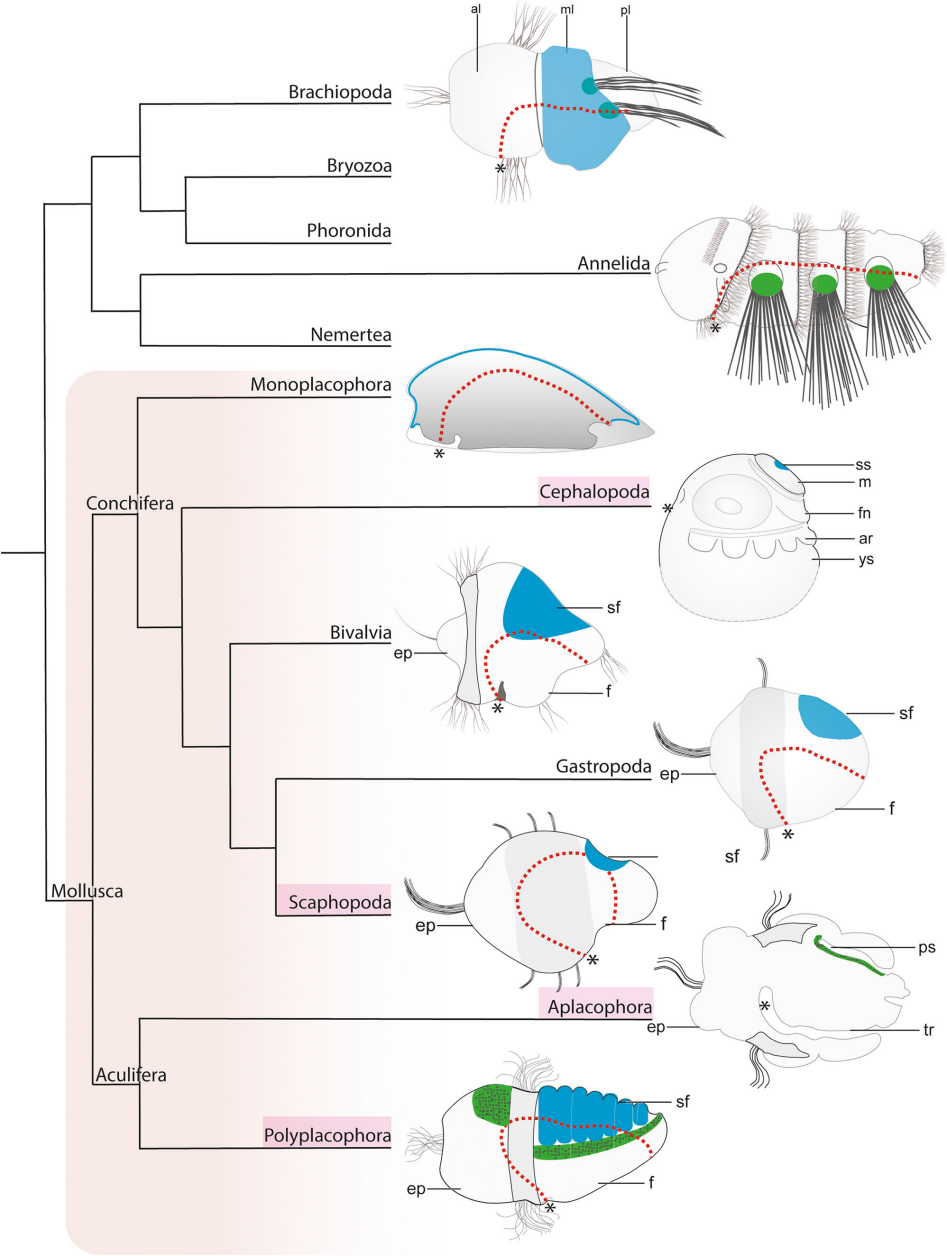
The distribution of shell fields (blue) and spicule‐bearing cells or chaetal sacs (green) in selected developmental stages of lophotrochozoans. Molluscan clades studied in this publication are highlighted in magenta. Branch lengths do not reflect phylogenetic distance or the relative evolutionary origin of taxa. Molluscan prototrochs are shaded in the dark gray, the mouth openings are labeled with asterisks, and the digestive tracts are labeled with red stippled lines. The trilobed larva of *Terebratalia transversa* is composed of an apical lobe (al), a mantle lobe (ml), and a pedicle lobe (pl). Chaetae are secreted by the chaetal sacs. The mid‐nectochaete larva (4 dpf) of the annelid *Platynereis dumerilii* exhibits chaetal sacs that secrete chaetae. An adult monoplacophoran is shown since developmental stages have not been investigated in detail. Cephalopods do not possess larvae sensu stricto. The early decapod cephalopod embryo exhibits an internalized shell sac (ss) that is situated below the mantle (m) on the dorsal side. The bivalve veliger larva exhibits two shell fields (sf) in the dorso‐lateral posttrochal region. The gastropod trochophore larva exhibits a shell field that secretes the shell. In some gastropod taxa, a veliger larva follows the trochophore larva during ontogeny. The trochophore larva of Scaphopoda forms a shell field on the dorsal side posterior to the prototroch. The pericalymma (test‐cell) larva of the Neomeniomorpha possesses a peri‐imaginal space (ps) that gives rise to the outgrowing trunk (tr). The latter is lined with spicule‐bearing cells. The late trochophore larva of polyplacophorans exhibits seven shell fields that are situated on the dorso‐lateral sides. The posterior‐most eighth shell field forms approximately a month after metamorphosis and settlement. Phylogeny adapted from Laumer et al. (2019) and Kocot et al. (2020). ar, arm, ep, episphere; f, foot; fn, funnel; ys, yolk sac.

Shells, spicules, opercula, gladii (pens), chaetae, or setae are hard parts that witness the rich diversity of fossil mollusks, brachiopods, annelids, and probably other lophotrochozoans that once roamed the earth (Zhang et al. 2013). Related structures are still present in recent lophotrochozoans and serve different functions such as protection against predators or the environment (e.g., desiccation), internal skeletons, buoyancy, anchorage in the substratum, or even as boring device (in shipworms) (Figure 1, Bezares‐Calderón et al. 2018; Thiel et al. 2017; Wanninger and Wollesen 2015).

In particular, the speciose molluscan clade is known for its diversity in number and anatomy of shells, valves, plates, and spicules and constitutes a phylogenetically informative clade that allows testing hypotheses about homology and evolutionary convergence (Figure 1, Wanninger and Wollesen 2015; Cavallo et al. 2022; Barrera Grijalba et al. 2023). While gastropods, cephalopods, scaphopods, and monoplacophorans possess a single shell in their ground plan, bivalves exhibit two shell valves, and polyplacophorans eight (Figure 1). In addition, aculiferans such as polyplacophorans and the aplacophoran neomeniomorphs and chaetodermomorphs possess spicules/sclerites that cover their girdle (perinotum) or the entire mantle, respectively (Figure 1; Wanninger and Wollesen 2015). The adult conchiferan shell is mainly composed of calcium carbonate, and most gastropods and bivalves possess a three‐layered shell composed of a periostracum, a prismatic, and a nacreous layer (Lowenstam and Weiner 1989; Checa et al. 2014). The periostracum is the outermost uncalcified shell layer, while the underlying shell layers are calcified and consist of aragonite and/or calcite polymorphs (Checa et al. 2014). Conchiferan shells develop via an invagination process of the mantle ectoderm, which gives rise to a crystallization chamber (Kniprath 1981). At the margins of this chamber, the periostracum starts to form and acts as a matrix for the subsequent secretion of minerals (Kniprath 1977, 1981; Berent et al. 2023). In conchiferans with an external shell, this crystallization chamber subsequently flattens, evaginates, and the shell is secreted (Kniprath 1981). Cell lineage studies show that in the polyplacophoran *Chaetopleura*, distinct cells contribute to shell fields (2d, 3c,d) and to the spicules (1a,d, 2a,c, and 3c,d) (Henry et al. 2004). Although the micromere 2d contributes to the shell fields of all mollusks studied so far, in polyplacophorans 3c and 3d contribute to the latter structure, while in conchiferans, other micromeres contribute (Wanninger and Wollesen 2015 for review). While conchiferan shells are composed of both aragonite and calcite, the shell plates of polyplacophorans are primarily composed of aragonite (with few calcite integrated into the shell plates) (Checa et al. 2017). Situated between cellular ridges on the dorsal side, the shell fields are formed in middle‐stage trochophore larvae (Figure 1) (Kniprath 1980). Two different cell types constitute the cellular ridge and probably secrete the cuticle that covers all shell fields (Kniprath 1980). Another two cell types constitute the shell fields that probably secrete the shell plates. The marginal cells develop large flat microvilli (stragulum) that insulate the shell field from the surrounding seawater. In these seven crystallization chambers, the shell plates are secreted (the eighth shell field forms approximately a month after settlement). In subsequent developmental stages, the margins of the shell plates are protected by the stragula. The presence of a periostracum, as seen in conchiferans, has been a subject of debate. While it is absent in polyplacophoran larvae, Haas et al. (1979) suggested that a so‐called “properiostracum” may develop later. In contrast, Kniprath (1980) found no evidence of a periostracum during early shell formation in polyplacophorans. In this context, Checa et al. (2017) proposed a homology between the cuticle‐producing girdle mantle epithelium of adult polyplacophorans and the periostracum‐secreting inner side of the outer mantle fold in adult bivalves. In polyplacophorans, the tegmentum as outermost partly organic layer of the shell plates is produced first (probably by the stragulum) and after flattening of the shell fields, the hypostracum is secreted as a second layer. All secreted shell plates are surrounded by a girdle of cells. These cells secrete spicules before the individual shell valves are secreted. Spicules are composed of aragonite and may vary significantly in their anatomy (Leise 1984; Leise and Cloney 1982). During the polyplacophoran shell field formation the typical invagination—evagination process as observed for conchiferans appears to be lacking. These differences in the formation of shell fields lead some authorities to suggest that polyplacophoran shell plates are not homologous to the ones of conchiferans (Scheltema 1993; Haas 1981; Eernisse and Reynolds 1994; Furuhashi et al. 2009; Kocot et al. 2016 for review). In the aculiferan neomeniomorphs, spicules are secreted by cells covering the trunk that grows out of the peri‐imaginal space (Figure 1).

To infer homologies between molluscan shells, it is crucial to distinguish between the different larval shell types and adult shells since they are secreted at different time points during ontogeny (Wanninger and Wollesen 2015 for review). For example, the prodissoconch I of bivalves is often followed by a teleoconch, that is, the adult shell. In some gastropods and bivalves, a third shell type is secreted, the protoconch II or prodissoconch II, respectively, while polyplacophorans only secrete a single shell plate type that is continuous into adulthood. The vast majority of coleoid cephalopods internalized and even reduced their shells to a degree that they are no longer visible. Those cephalopods that still secrete a shell do not exhibit an embryonic shell in contrast to many gastropods. Studies on conchiferan gastropods and bivalves demonstrated that the tissue secreting the nacre layer of adult shells has very different transcriptomic interspecific profiles (secretomes) and may therefore not be homologous (e.g. Jackson et al. 2010). On the contrary, for molluscan biomineralization four domains of shell matrix proteins appear to be critical, that is, carbonic anhydrase, chitin‐binding, von‐Willebrand‐factor A, and tyrosinase (Arivalagan et al. 2017; Cavallo et al. 2022). Moreover, comprehensive studies on a variety of molluscan representatives showed that the transcription factors *gbx* and *pax2/5/8* are expressed in the shell fields and spicule‐bearing cells in early developmental stages of various molluscan representatives (Wollesen et al. 2015a, 2017).

To shed light on the gene expression inventory expressed in the developing molluscan mantle, we investigated candidate genes known to be expressed in the gastropod shell field. *Goosecoid* (*gsc*), *grainyhead* (*grh*), *homeobox 1* (*hox1*), *notch*, *zinc finger protein of the cerebellum* (*zic*), *chitin‐synthase* (*chs*), and *ferritin* (*fer*) were studied in representative molluscan clades such as the aculiferan polyplacophoran *Acanthochitona fascicularis* (Linnaeus, 1767), the aplacophoran neomeniomorph *Wirenia argentea* Odhner, 1921, the conchiferan scaphopod *Antalis entalis* Linnaeus 1758, and the conchiferan cephalopod *Xipholeptos notoides* (Berry, 1921). In model organisms, the transcription factor encoding gene *goosecoid* has been shown to play a role in the regulation and migration of cells during gastrulation (Blum et al. 1994), while *grainyhead* is a transcription factor encoding gene that is essential for the differentiation of exocrine cells (Yamaguchi et al. 2006). The receptor *notch* and its ligand *delta* are key players of a highly conserved cell signaling pathway. They are involved in various developmental processes, including cell fate determination, differentiation, proliferation, and apoptosis (Bray 2006). *Zic* encodes a transcription factor and is involved in early embryogenesis, particularly in neurogenesis (Grinberg and Millen 2005). *Hox1* is a homeobox gene which is expressed in a staggered fashion together with other *hox* genes along the anterior‐posterior axis of many bilaterians (Wollesen et al. 2018; Wollesen and Wanninger 2023 for review). *Chitin synthase* is an enzyme synthesizing chitin, a structural polysaccharide among others involved in the secretion of the chitinous matrix of the gastropod shell (Schönitzer and Weiss 2007). *Fer* has been shown to be expressed in the shell fields of gastropods as well as brachiopods (Jackson et al. 2006; Wernström et al. 2022). All molluscan representatives studied herein follow different shell and spicule secretion strategies during their ontogeny, resulting in differently shaped and mineralized adult hard parts. Nevertheless, nearly all of the above‐mentioned candidate genes are expressed in the region of the shell fields, as well as the spicule‐bearing cells, suggesting that these morphological structures share a deep evolutionary history.

## 2 Methods

### 2.1 Collection and Culture of Animals

In the summer of 2012, adult specimens of the polyplacophoran *Acanthochitona fascicularis* were collected from the intertidal zone near the Station Biologique Roscoff (Roscoff, France). Similarly, adult *Antalis entalis* were collected from a depth of approximately 15 m via dredging, conducted by the crew of the *Neomys* (Station Biologique de Roscoff), off the coast of Roscoff. Both species were transferred to dishes with seawater and maintained at a temperature of 18°C. Spawning occurred naturally, after which the eggs were repeatedly rinsed in seawater and fertilized using sperm. The lecithotrophic developmental stages were cultured in glass dishes with daily water changes, and metamorphosis was induced by adding shell gravel encrusted with *Lithothamnion* species.

Adults of the pygmy squid *Xipholeptos notoides* (formerly referred to as *Idiosepius notoides*) were dip‐netted in the sea grass beds of Moreton Bay, Queensland, Australia. Developmental stages were cultured and staged as described previously (Yamamoto 1988; Wollesen et al. 2014).

Adults and larvae of the neomeniomorph *Wirenia argentea* were collected and cultured as described previously (Redl et al. 2014, 2016, 2018). In brief, individuals were collected, maintained, and reared during the periods from January to May 2012, November 2012 to February 2013, and November to December 2013, respectively. Sediment samples were kept in 20 μm‐filtered, UV‐sterilized seawater with a salinity of 35‰ (FSSW) that had been pre‐cooled to 4°C. Every 4 days, adult specimens were transferred to clean plastic jars with fresh FSSW, which increased egg‐laying productivity. The freshly laid eggs were then moved to clean plastic jars containing FSSW and kept under the same conditions as the adults. After hatching, *Wirenia* undergoes development via the pericalymma or test cell larval stage. The age of the larvae is provided in days post‐hatching (dph). We identified four morphologically distinct stages: freshly hatched test cell larva (0–1 dph), early test cell larva (6–7 dph), mid‐stage test cell larva (10–11 dph), and late test cell larva (14–16 dph), with each stage encompassing a developmental time range of approximately 24 h.

### 2.2 RNA Extraction and Fixation of Animals for In Situ Hybridization Experiments

For all four species, several hundred individuals of different developmental stages were fixed for in situ hybridization experiments and used for RNA extraction as previously described (Redl et al. 2016, 2018; Wollesen et al. 2014, 2018).

### 2.3 Alignment and Orthology Analysis

Amino acid sequences of the genes *gsc*, *grh*, *chs*, *fer*, *notch*, *zic*, and *hox1* of various metazoan species were retrieved from GenBank (accession numbers provided in Supporting Information Table 1). These sequences were used in BLAST [1] (Camacho et al. 2009) searches against the assembled and published transcriptomes of *Acanthochitona fascicularis, Antalis entalis, Xipholeptos notoides*, and *Wirenia argentea* (De Oliveira et al. 2016). Predicted amino acid sequences of candidate genes were aligned with their putative metazoan orthologs using Clustal Omega and then trimmed using ClipKit v21.2.3 (Steenwyk et al. 2020). The clipped alignments were manually reviewed with Jalview v2.11.3.3 (Waterhouse et al. 2009). Afterwards, the amino acid substitution models were estimated using Prottest3 v3.4.2 (Darriba et al. 2011) based on the AIC criterion (models used for each gene are provided in supplementary table 3). Orthology analyzes were carried out based on a Bayesian analysis with MrBayes v3.2.7 (Ronquist et al. 2012) for one million generations, sampling every 1000 generations with 16 chains and burn‐in of 25% of trees. The resulting trees were manually rooted and visualized using FigTree v1.4.5 (FigTree. http://tree.bio.ed.ac.uk/software/figtree/; accessed Sep 2024).

### 2.4 Molecular Isolation of RNA Transcripts

First‐strand cDNA synthesis of the RNA pooled from different developmental stages of *Acanthochitona fascicularis* was carried out by reverse transcription using the First strand cDNA Synthesis Kit for rt‐PCR (Roche Diagnostics GmbH, Mannheim, Germany for the experimental procedure). Identified orthologous sequences were used to design gene‐specific primers and PCR products were size‐fractioned by gel electrophoresis; gel bands of the expected lengths were excised and cleaned up using a QIAquick Gel Extraction Kit (QIAgen, Hilden, Germany). Cleaned‐up products were cloned by insertion into pGEM‐T Easy Vectors (Promega, Mannheim, Germany). Plasmid minipreps were grown overnight, cleaned up with the QIAprep Spin MiniprepKit (QIAgen), and sent off for sequencing. Sequences were identified using the BLASTx algorithm screening the respective transcriptomes (De Oliveira et al. 2016). All nucleotide sequences, as well as their deduced amino acid sequences, were submitted to GenBank (Accession numbers: PQ360976‐PQ360992).

### 2.5 Probe Synthesis and Whole‐Mount in Situ Hybridization

Riboprobe templates were amplified via standard PCR from isolated plasmids using M13 forward and reverse primers. In vitro transcription reactions were performed with these templates, digoxigenin‐UTP (DIG RNA Labeling Kit, Roche Diagnostics), and SP6/T7 polymerase (Roche Diagnostics GmbH) for the syntheses of antisense riboprobes, according to the manufacturer's instructions. In whole‐mount in situ hybridization experiments, specimens were rehydrated into PBT (phosphate buffered saline + 0.1% Tween‐20) and treated with Proteinase‐K at 37°C for 10 min. Developmental stages of *Acanthochitona fascicularis* were Proteinase‐K treated with 55 µg/mL in PBT, those of *Antalis entalis* with 45 µg/mL, the one of *Xipholeptos notoides* with 25 µg/mL, and those of *Wirenia argentea* with 10 µg/mL. Specimens were prehybridized in hybridization buffer for 4 h either at 62°C (*A. fascicularis*), 50°C (*A. entalis*), 65°C (*X. notoides*), or 56°C (*W. argentea*). Hybridization was performed at the same temperature with probe concentrations ranging between 0.5 and 1 μg/ml for 21–24 h. A DIG‐labeled AP‐antibody was used at a dilution of 1:5000 in blocking solution at 4°C overnight. Color development in the NBT/BCIP/Alkaline Phosphatase buffer solution took 6–24 h at 4°C. For counterstains of cell nuclei, sections were stained with DAPI (Sigma‐ Aldrich, St. Louis, MO) and washed in phosphate‐buffered saline. The majority of whole‐mount preparations were cleared in a solution of benzyl‐benzoate and benzyl alcohol, mounted on objective slides, and analyzed. Preparations were documented with an Olympus BX53 Microscope (Olympus, Hamburg, Germany). In addition, developmental stages were scanned with a Leica confocal SP5 II microscope (Leica Microsystems, Wetzlar, Germany) using bright‐field, autofluorescence, and reflection mode scans to understand the precise location of transcripts. If necessary, images were processed with Adobe Photoshop 9.0.2 software (San Jose, CA) to adjust for contrast and brightness. Sketch drawings were created with Adobe Illustrator CC 2015.1.0 (Adobe Systems Inc., San Jose, CA).

## 3 Results

### 3.1 Phylogenetic Analyzes of Candidate Genes

The phylogenetic analyzes on the candidate genes *grh, gsc, chitin‐synthase, fer, notch, delta, zic* confirmed their identity (Supporting Information Figures 1–6; Supplementary Information Table 2). The identity of *hox1* of the neomeniomorph *Wirenia argentea* was confirmed in line with a previous study (De Oliveira et al. 2016) but gene expression was first visualized in the present study.

### 3.2 *Hox1*‐expression

**Figure 2:**
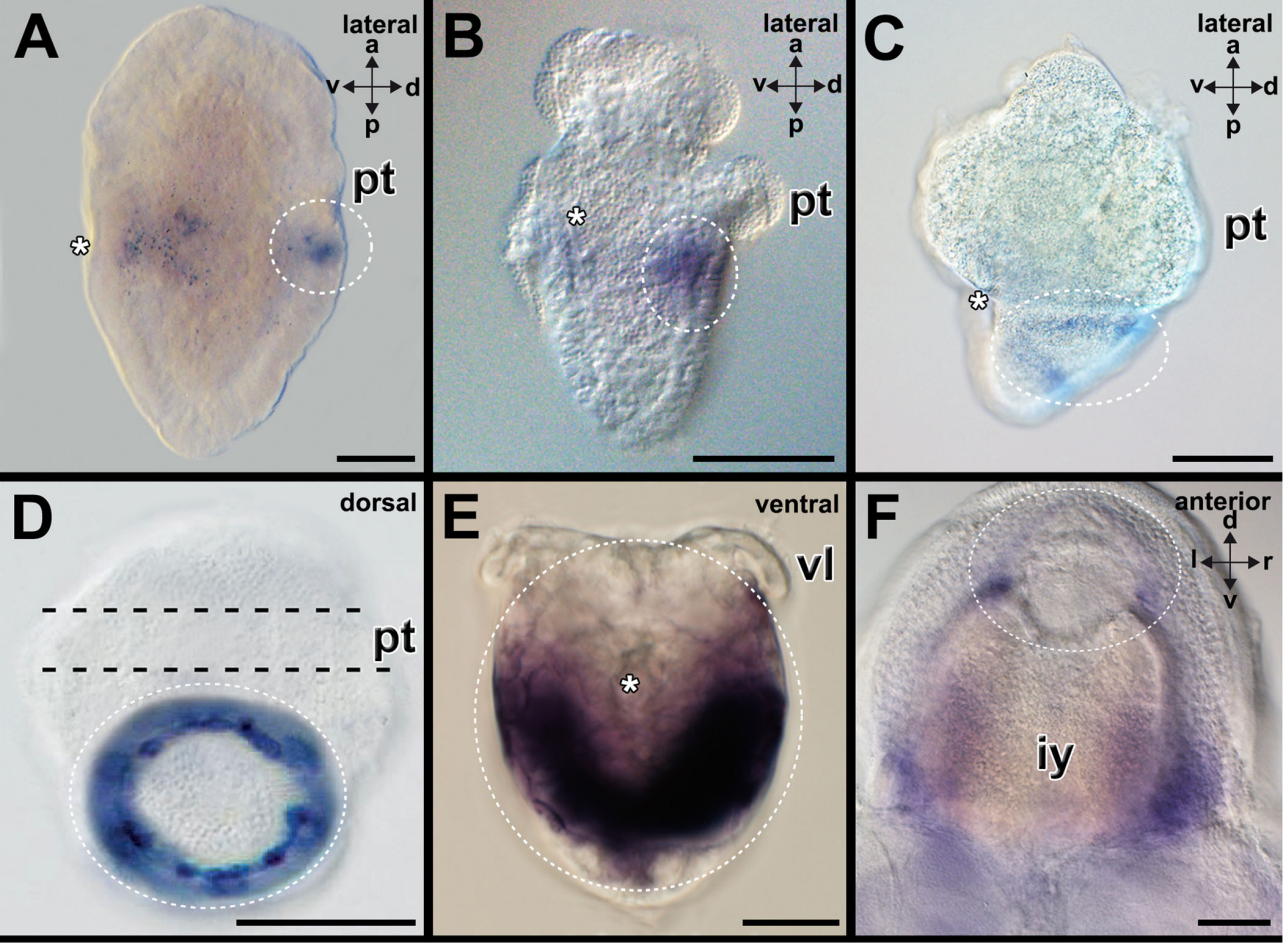
*Hox1* is expressed by the spicule‐bearing cells and in the shell fields of mollusks. Dorsal (d)‐ventral (v), anterior (a)‐posterior (p), and left (l)‐right (r) axes indicate the orientation. Asterisks mark the region of the mouth. (A) The trochophore larva of the polyplacophoran *Acanthochitona fascicularis* expresses *hox1* in the first shell field (encircled) on the dorsal side. (B) The pericalymma larva (test‐cell larva) of the neomeniomorph aplacophoran *Wirenia argentea* expresses *hox1* lining the peri‐imaginal space of the outgrowing trunk (only dorsal side encircled). (C) The trochophore larva of the scaphopod *Antalis entalis* expresses *hox1* in outermost cells of the shell field (encircled) on the dorso‐lateral side. (D) The trochophore larva of the gastropod *Gibbula varia* expresses *hox1* in the outermost cells of the shell field (encircled) on the dorsal side. (E) The veliger larva of the bivalve *Dreissena polymorpha* expresses *hox1* in cells of the shell fields (encircled) on the dorso‐lateral sides. (F) The embryo of the cephalopod *Octopus vulgaris* expresses *hox1* in the shell sac (encircled) (see Barrera Grijalba et al. 2023 for details). Images A, D, and E courtesy of Martin Fritsch (Berlin), Gerhard Steiner (Vienna), and David A. Salamanca‐Díaz (Exeter), respectively. See Supporting Information Figure 7 for expression pattern on other developmental stages. iy, internal yolk; pt, prototroch; vl, velar lobe. Scale bars: 50 µm (except C and F: 100 µm, D: 25 µm, E: 20 µm).

*Hox1* has been shown to be expressed in polyplacophoran as well as conchiferan mollusks in previous studies (Figure 2A,C,D–F). In the aplacophoran mollusk *Wirenia argentea*, *hox1* is expressed in spicule‐bearing cells that line the tissue surrounding the peri‐imaginal space of 16 days post‐hatching (dph) larvae, while earlier developmental stages, such as 6–7 dph or 10–11dph old larvae did not exhibit any staining (Figure 2B; Supporting Information Figure 7).

### 3.3 *Gsc*‐expression

**Figure 3:**
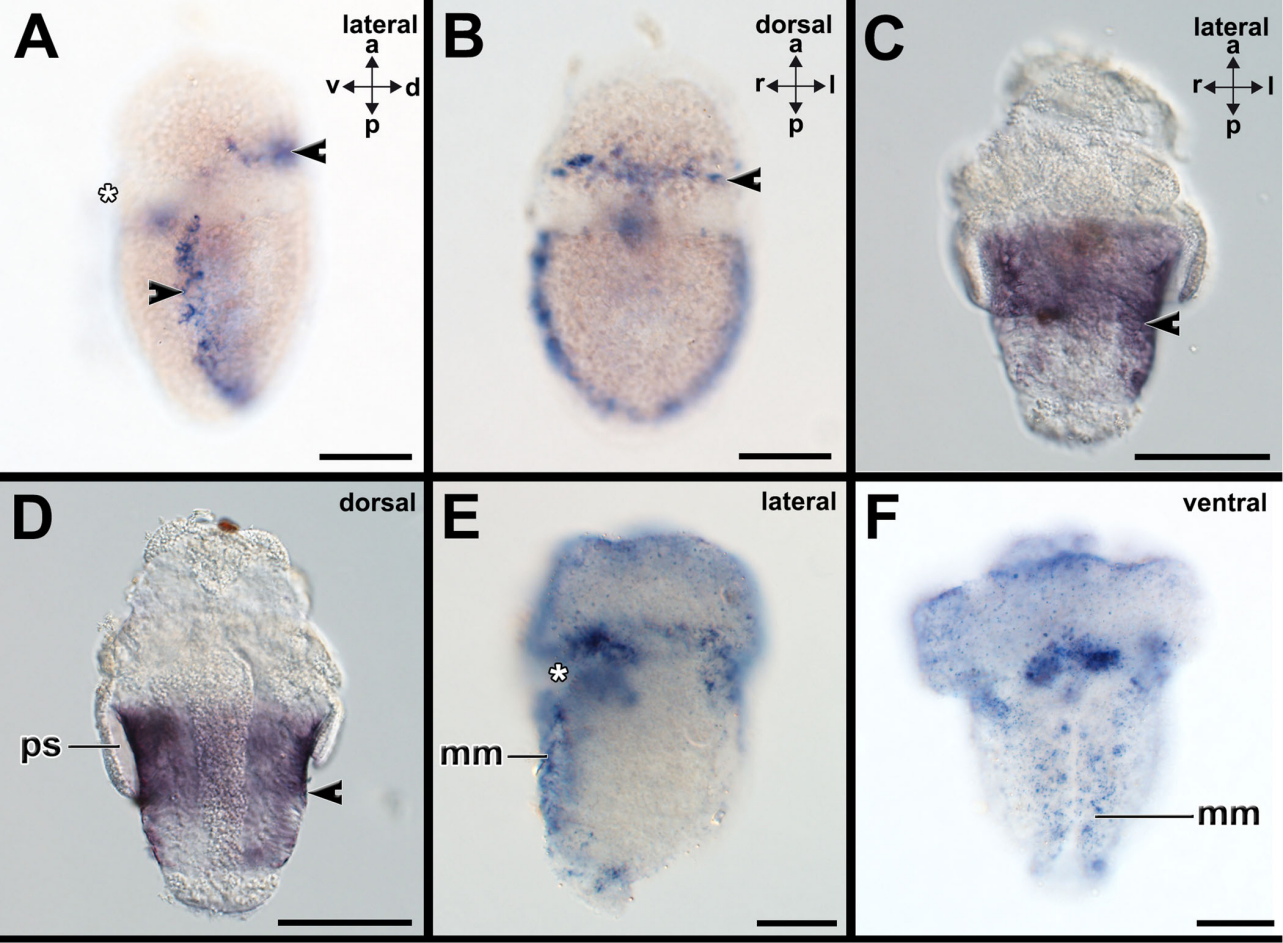
*Goosecoid* (*gsc*) is expressed in spicule‐bearing cells and the shell field. Dorsal (d)‐ventral (v), anterior (a)‐posterior (p), and left (l)‐right (r) axes indicate the orientation. Asterisk marks the region of the mouth. (A and B) The trochophore larva of the polyplacophoran *Acanthochitona fascicularis* expresses *gsc* in spicule‐bearing cells (arrowheads) of the girdle on the dorsal and lateral sides. (C and D) The pericalymma larva (test‐cell larva) of the neomeniomorph aplacophoran *Wirenia argentea* expresses *gsc1* in spicule‐bearing cells (arrowheads) lining the peri‐imaginal space (ps) of the outgrowing trunk. *Gsc1* and *gsc2* are expressed in similar expression domains in the neomeniomorph pericalymma larva (c.f. Supporting Information Figures 9, 10). (E and F) The trochophore larva of the scaphopod *Antalis entalis* expresses *gsc* along the entire mantle margin (mm) and in the region surrounding the mouth (asterisk). See Supporting Information Figures 8–11 for expression patterns on other developmental stages. Scale bars: 50 µm.

Early trochophore larvae of the polyplacophoran *Acanthochitona fascicularis* express *gsc* in the spicule‐bearing cells of the forming pretrochal and posttrochal girdle (Supporting Information Figure 8A–C). The spicule‐bearing cells in the hyposphere are distributed from the lateral sides to the dorsal side and form almost a continuous circle around the trochophore larva (Supporting Information Figure 8A–C). *Gsc* is also expressed anterior to the mouth opening and in a small domain in the posterior dorsal region that may correspond to the posterior‐most girdle (Supporting Information Figure 8A,C). Middle‐stage and late‐stage trochophore larvae express *gsc* in the spicule‐bearing cells of the girdle and around the mouth opening (Figure 3A,B; Supporting Information Figure 8D–I). In the latter developmental stages, *gsc+* spicule‐bearing cells are present in the entire girdle that is still divided by the prototroch. In the neomeniomorph *Wirenia argentea* two different *gsc* genes were identified and gene expression patterns visualized (Figure 3C,D; Supporting Information Figures 9A–H, 10A–C). *Gsc1* is expressed in the cells that line the tissue surrounding the peri‐imaginal space of 6–7 dph and 16 dph old pericalymma larvae (Figure 3C,D; Supporting Information Figure 9A–F). Subsequent developmental stages exhibit *gsc1+* cells covering the trunk, while the posterior‐most telotroch does not show *gsc1+* cells (Supporting Information Figure 9G,H). *Gsc2* is expressed in the cells that line the tissue surrounding the peri‐imaginal space of 6–7 dph (Supporting Information Figure 10A–C).

In the conchiferan scaphopod *Antalis entalis gsc* is expressed in a paired domain in the forming shell field and in the region around the mouth of early stage trochophores (Supporting Information Figure 11A–C). In mid‐stage trochophores *gsc* is expressed along the entire mantle margin and in the region surrounding the mouth (Figure 3E,F; Supporting Information Figure 11D–F). Late mid‐stage trochophores express *gsc* around the mouth and along the anterior mantle margin (Supporting Information Figure 11G‐I).

### 3.4 *Grh*‐expression

**Figure 4:**
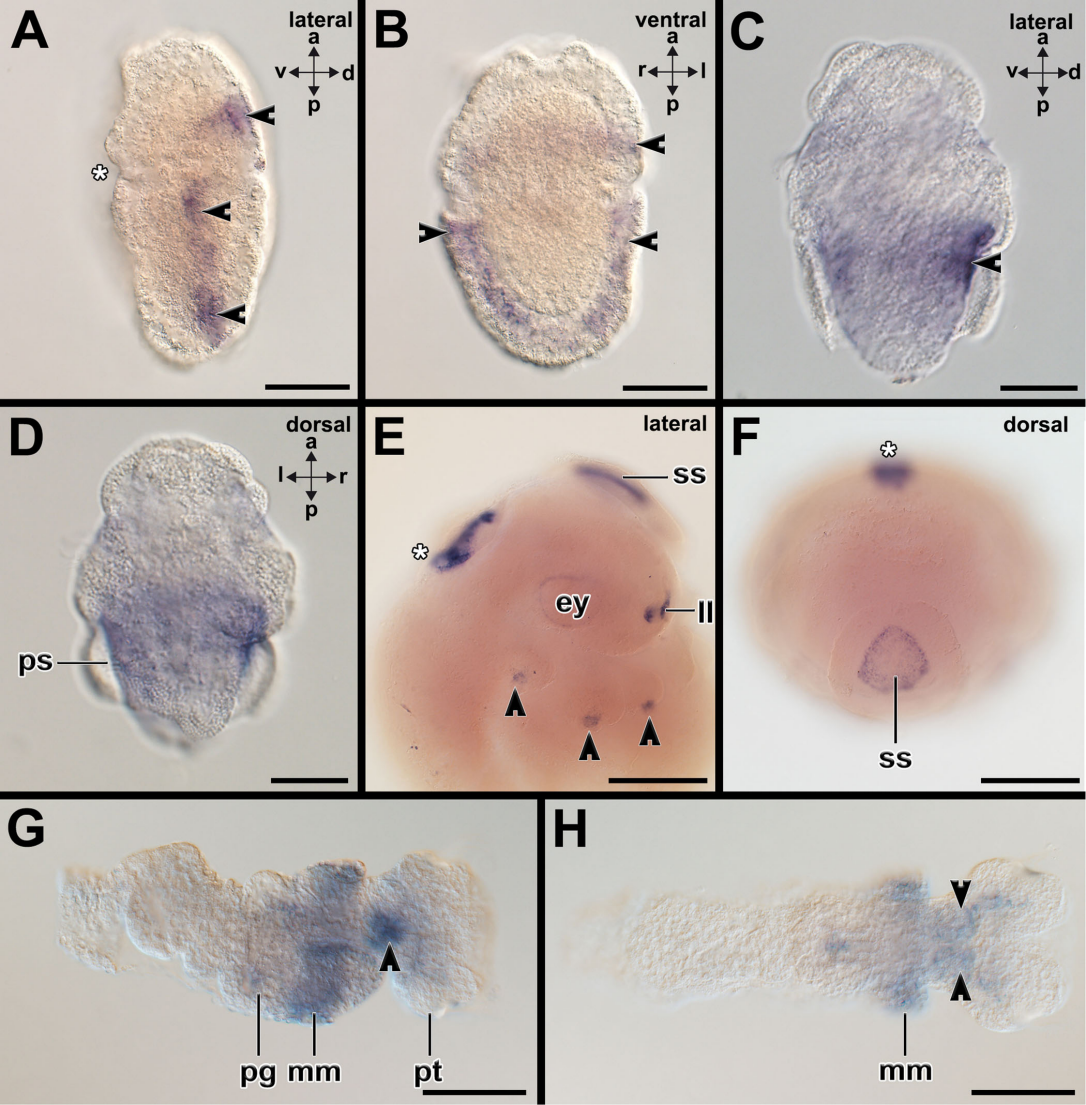
*Grainyhead* (*grh*) is expressed in spicule‐bearing cells and the shell field. Dorsal (d)‐ventral (v), anterior (a)‐posterior (p), and left (l)‐right (r) axes indicate the orientation. Asterisks mark the region of the mouth. (A and B) Late‐stage trochophore larvae express *grh* in the spicule‐bearing cells (arrowheads) of the anterior and lateral girdle. The posterior‐most domain of the girdle only houses few *grh*‐expressing cells. (C and D) In early stage test cell larvae of the neomeniomorph *Wirenia argentea* (6–7 dph) *grh* is expressed in the spicule‐bearing cells that line the tissue surrounding the peri‐imaginal space (ps) and the outgrowing trunk. (E and F) In the early developmental stages (stage 19) of the cephalopod *Xipholeptos notoides grh* is expressed in the region of the shell sac (ss), the median regions of the developing arms, where the suckers develop (arrowheads), the mouth region (asterisk) where the jaw develops, and the region of the lateral lips (ll) (posterior to the eyes (ey)). Slight expression is also visible in developing eyes. (G and H) Late‐stage trochophores express *grh* in their anterior mantle margin (mm) as well as in the region of the cerebral ganglia and/or the apical organ (region marked with arrowheads), and pedal ganglia (pg) as well as the prototroch (pt) that is in the process of degeneration. See Supplementary Information Figures 12–15 for expression patterns on other developmental stages. Scale bars: A–D: 50 µm, E–F: 150 µm, G–H: 100 µm.

Early trochophore larvae of the polyplacophoran *A. fascicularis* express *grh* in the posterior‐most region of the forming girdle, in addition to a domain anterior to the prototroch where the anterior girdle forms (Supporting Information Figure 12A–C). Weak expression is also visible in epidermal cells of the lateral episphere (arrowheads in Supporting Information Figure 12A,B). Middle‐stage and late‐stage trochophore larvae express *grh* in the spicule‐bearing cells of the anterior and lateral girdle (Figure 4A,B; Supporting Information Figure 12D–I). The posterior‐most domain of the girdle only houses few *grh*‐expressing cells (Figure 4B). In early‐stage test cell larvae of the neomeniomorph *Wirenia argentea* (6–7dph) *grh* is expressed in the spicule‐bearing cells that line the tissue surrounding the peri‐imaginal space and the outgrowing trunk (Figure 4C,D), while 16dph old embryos only faintly express *grh* in that region (Supporting Information Figure 13). In the early developmental stages (stage 19) of the cephalopod *Xipholeptos notoides grh* is expressed in the region of the shell gland, the median regions of the developing arms, where the suckers develop, the mouth region where the jaw develops, and the region of the lateral lips (posterior to the eyes) (Figure 4E,F; Supporting Information Figure 14A–C). Slight expression is also visible in developing eyes (Figure 4E). More advanced developmental stages (stage 25) express *grh* in the suckers of the arms, the region of the mouth with the jaw, and the adjacent esophagus (Supporting Information Figure 14D,F). Additional staining is visible in the developing eyes (Supporting Information Figure 14F). No *grh*‐expression was detected in subsequent developmental stages (stage 28) (Supporting Information Figure 14G,H). In early trochophore larvae of the scaphopod *Antalis entalis grh* is expressed in few cells of the forming shell field, the region of the forming pavilion and the lateral anterior foot and mouth (Supporting Information Figure 1). *Grh*+ cells are also present in a region posterior to the cerebral pits in which in a previous study, *elav*+ cells were found (c.f. Supporting Information Figure 15B,C with Figure S1 in Wollesen et al. 2018). In mid‐stage trochophore *grh* is expressed in few cells surrounding the foot and in the region of the forming pedal ganglia (anterior to statocysts) (c.f. Wollesen et al. 2018, *elav* expression) (Supporting Information Figure 15D–F). Late‐stage trochophores express *grh* in their anterior mantle border as well as in the region of the cerebral ganglia, the apical organ, and pedal ganglia as well as the prototroch that is in the process of degeneration (Figure 4G,H).

### 3.5 *Chs*‐expression

**Figure 5:**
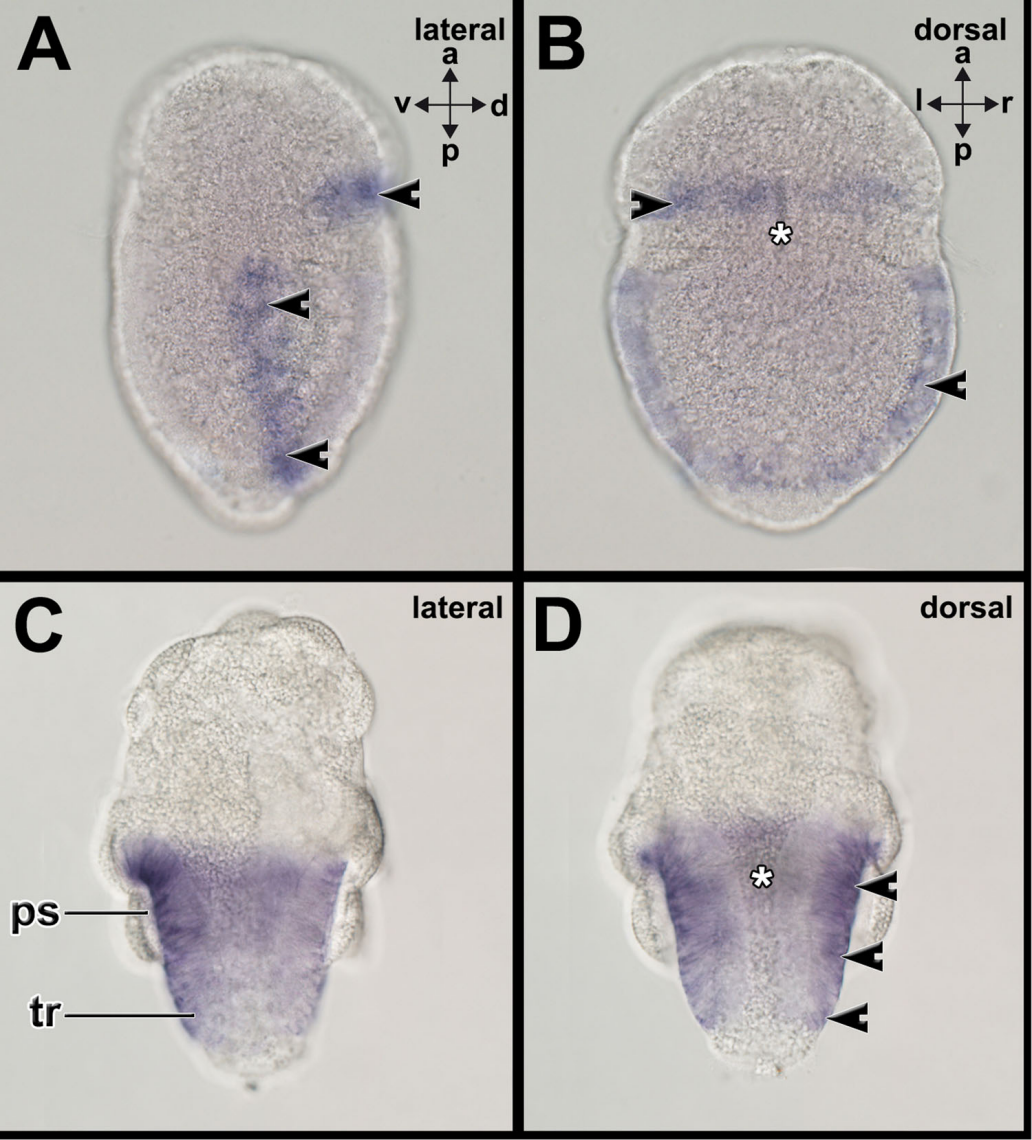
*Chitin synthase* is expressed in the spicule‐bearing cells of aculiferan mollusks. Dorsal (d)‐ventral (v), anterior (a)‐posterior (p), and left (l)‐right (r) axes indicate the orientation. Asterisks mark the foregut. (A and B) Middle‐stage and late‐stage trochophore larvae of the polyplacophoran *Acanthochitona fascicularis* express *chs* in the spicule‐bearing cells (arrowheads) of the girdle. (C and D) In late‐stage larvae (16 hph) of the neomeniomorph *Wirenia argentea*, *chs* is expressed in the spicule‐bearing cells (arrowheads) that line the tissue surrounding the peri‐imaginal space (ps). In late‐stage larvae *chs+* cells are lining the epidermis of the trunk (tr). The posterior‐most region of the trunk does not exhibit *chs+* cells. See Supplementary Information Figures 16 and 17 for expression patterns on other developmental stages. Scale bars: 50 µm.

No *chs*‐expression was observed in the early trochophore larva of the polyplacophoran *A. fascicularis* (Supporting Information Figure 16A–C). Middle‐stage and late‐stage trochophore larvae express *chs* in the spicule‐bearing cells of the girdle (Figure 5A,B; Supporting Information Figure 16D–I). In mid‐stage test cell larvae (6–7 dph) and late‐stage larvae 16 dph) of the neomeniomorph *Wirenia argentea*, *chs* is expressed in the spicule‐bearing cells that line the tissue surrounding the peri‐imaginal space (Figure 5C,D; Supporting Information Figure 17A–F). In late‐stage larvae *chs+* cells are lining the epidermis of the trunk. The posterior‐most region of the trunk does not exhibit *chs+* cells (Figure 5C,D, Supporting Information Figure 17D–F).

### 3.6 *Fer*‐expression

**Figure 6:**
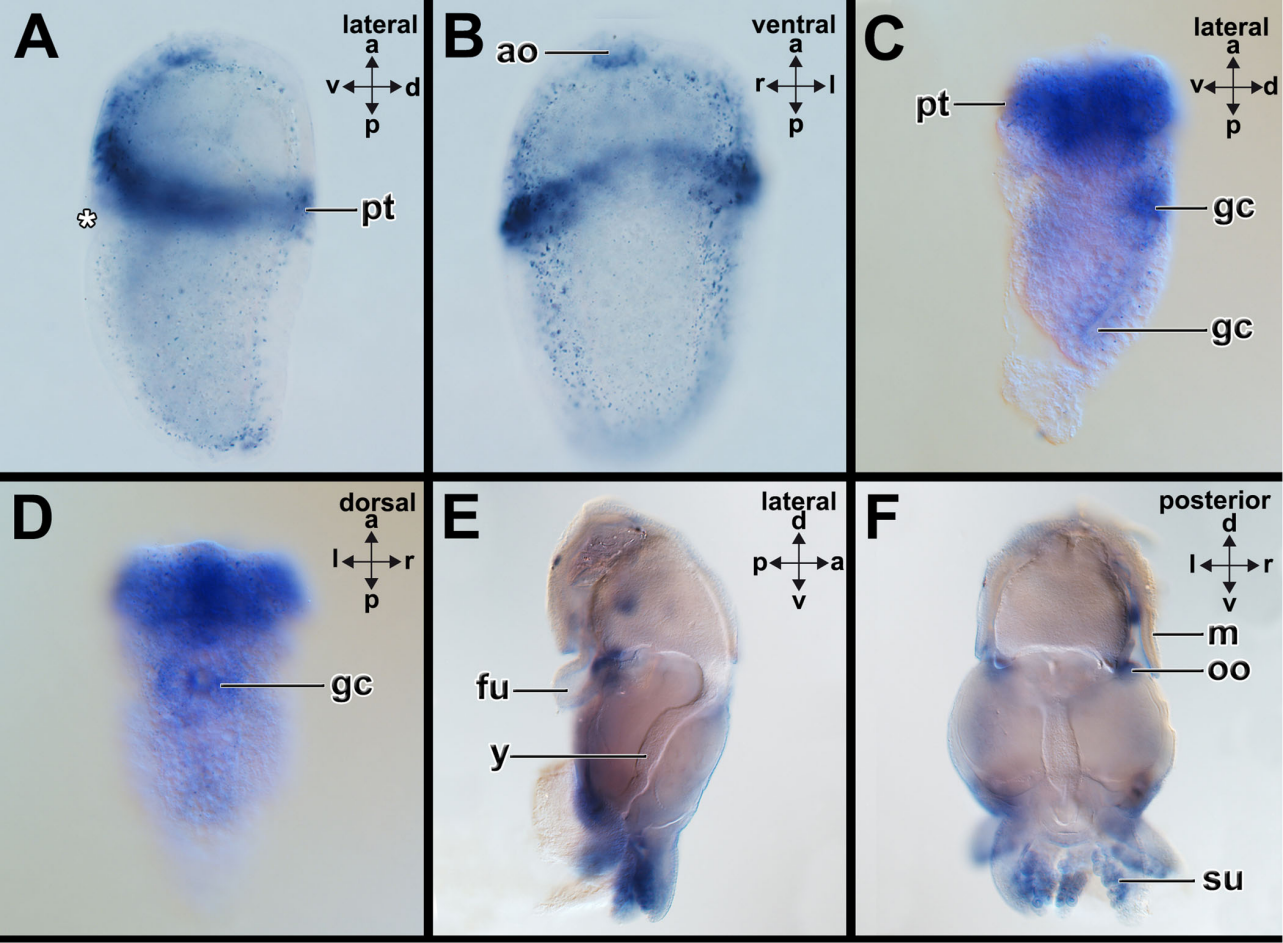
*Ferritin* is not expressed in the spicule‐bearing cells or in the shell fields of polyplacophorans, scaphopods, and cephalopods. Dorsal (d)‐ventral (v), anterior (a)‐posterior (p), and left (l)‐right (r) axes indicate the orientation. Asterisk marks the region of the mouth. (A and B) Late‐stage trochophore larvae of the polyplacophoran *Acanthochitona fascicularis* express *fer* in the cells of the prototroch (pt) and the apical organ (ao). (C and D) In mid‐stage trochophores of the scaphopod *Antalis entalis fer* is expressed in the prototroch and the glandular cells (gc) of the mid‐ and hindgut. (E and F) Developmental stage 25 individuals of the cephalopod *Xipholeptos notoides* express *fer* in the suckers (su), and the olfactory organ (oo). fu, funnel; m, mantle; y, yolk. See Supplementary Information Figures 18–20 for expression patterns on other developmental stages. Scale bars: A–B: 20 µm and C–F: 100 µm.

Early trochophore larvae of the polyplacophoran *A. fascicularis* express *fer* in cells of the apical organ and the prototroch (Supporting Information Figure 18A–C). *Fer* is also faintly expressed in the entire embryo (Supporting Information Figure 18A–C). Middle‐stage trochophores express *fer* in cells of the apical organ and few cells in the posterior‐most region (Supporting Information Figure 18D–F). Late‐stage trochophores express *fer* in the cells of the prototroch and the apical organ (Figure 6A–B; Supporting Information Figure 18G–I). In the scaphopod *A. entalis*, *fer* is expressed around the mouth in early‐stage trochophores (Supporting Information Figure 19A–C). Additional *fer+* cells are distributed along the ventral episphere (Supporting Information Figure 19A). *Fer* is faintly and globally expressed in early larvae. In mid‐stage trochophores, *fer* is expressed in the apical organ, the prototroch, and the glandular cells of the mid‐ and hindgut (Figure 6C,D; Supporting Information Figure 19D–F). Late‐stage trochophores express *fer* in the prototroch and the glandular tissue of the mid‐ hindgut (Supporting Information Figure 19G,H). In the cephalopod *X. notoides*, early developmental stage 19 expresses *fer* globally in the epidermal layers of various organ systems such as the mantle, the arms, the eyes, or the gills (Supporting Information Figure 1). In more advanced developmental stage 25, individuals express *fer* in the suckers and the olfactory organ (Figure 6E–F; Supporting Information Figure 20D–F). Stage 28 individuals express *fer* in the posterior regions of the animals (Supporting Information Figure 20G,H).

### 3.7 *Notch*‐expression

**Figure 7:**
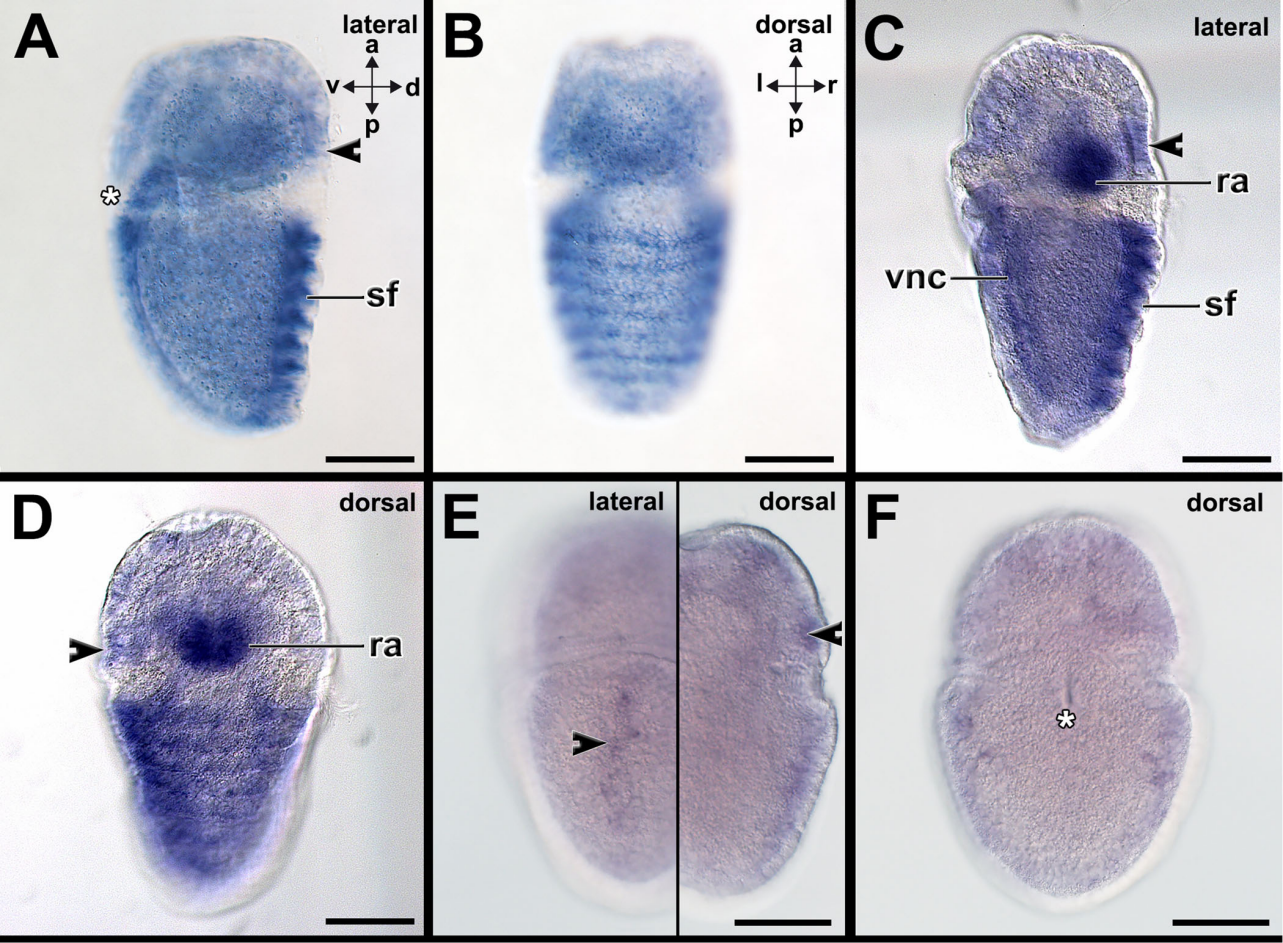
*Notch*, *delta*, and *zic* are expressed in the shell fields and spicule‐bearing cells of the polyplacophoran *Acanthochitona fascicularis.* Dorsal (d)‐ventral (v), anterior (a)‐posterior (p), and left (l)‐right (r) axes indicate the orientation. Asterisks mark the region of the mouth (A) or the foregut (F). (A and B) In late stage trochophore larvae *notch*‐expression is restricted to the shell fields (sf), spicule‐bearing cells (arrowheads), and the forming ventral nerve cord (not visible). (C and D) *Delta*‐expression is present in the shell fields (sf), the spicule‐bearing cells (arrowheads), and the forming ventral nerve cord (vnc) in late stage trochophore larvae. Additional expression is visible in the region around the forming radula (ra). (E and F) Middle stage trochophore larvae express *zic* in their spicule‐bearing cells (arrowheads) of the perinotum of the lateral hyposphere (E, left) and in spicule bearing cells of the episphere (E, right). In addition, *zic* is expressed in the apical organ, on the ventral side posterior to the mouth in the foot (E, right image; supplementary information Figure 23). See Supplementary Information Figures 21, 22, 23 for expression patterns on other developmental stages. Scale bars: A–B: 50 µm.

In early trochophores of *A. fascicularis*, *notch* is expressed in the ectodermal layer of the episphere, in the posterior‐most medium hyposphere, and adjacent to the prototroch in the hyposphere (Supporting Information Figure 21A–C). In middle trochophore larvae *notch*‐expression is found in the region of the forming ventral nerve cords and the shell fields (Supporting Information Figure 21D–F). In addition, *notch*‐expression is present posterior to the apical organ, i.e., the region of the forming cerebral commissure (Supporting Information Figure 21D,F). In late stage trochophore larvae expression is restricted to the ventral nerve cords, the shell fields, and spicules (Figure 7A,B; Supporting Information Figure 21G–I).

### 3.8 *Delta*‐expression

Early stage trochophore larvae of the polyplacophoran *A. fascicularis* express *delta* in two regions flanking the mouth opening (Supporting Information Figure 22A–C). Other *delta+* cells are present in ectodermal domains of the episphere. The dorsal side of the early trochophore larvae is devoid of *delta+* cells (Supporting Information Figure 22C). Mid‐stage trochophore larvae express *delta* in the region of the forming nerve cords in the ventral hyposphere and in ectodermal cells of the median lateral episphere (Supporting Information Figure 22D–F). Late stage polyplacophoran trochophore larvae of *Delta+* cells are present in the shell fields, the spicule‐bearing cells, the ventral nerve cords, and in the region of the anlage of the radula (Figure 7C–D; Supporting Information Figure 22G–I).

### 3.9 *Zic*‐expression

Early trochophore larvae of the polyplacophoran *A. fascicularis* express *zic* in the episphere, among others in the apical organ (Supporting Information Figure 23A–C). Few cells are present in the hyposphere in the region of the nascent foot but not in the region of the future shell fields (Supporting Information Figure 23A–C). *Zic*+ cells are located in the region that may give rise to the spicules (Supporting Information Figure 23A). Middle trochophore larvae express *zic* in their spicule‐bearing cells of the perinotum of the lateral hyposphere (Figure 7E–F; Supporting Information Figure 23D–F) and in spicule bearing cells of the episphere (Figure 7E). In addition, *zic* is expressed in the apical organ (Supporting Information Figure 23F (inset), in the ventral side posterior to the mouth in the foot (Figure 7E, right image). Late stage trochophore larvae express *zic* in the region around the apical organ, in few spicule‐bearing cells anterior to the prototroch, and in few cells of the shell fields (Supporting Information Figure 23G–I).

## 4 Discussion

### 4.1 Molluscan Shell Fields and Spicule‐bearing Cells Share the Same Transcription Factors

In this study, we present the gene expression patterns of transcription factors and effector genes across an unprecedented diversity of molluscan representatives during ontogeny. Unlike previous studies, which primarily focused on gastropods and bivalves, we demonstrate that these evolutionarily conserved genes are expressed not only in molluscan shell fields but also in spicule‐bearing cells.

For example, *lab* (*hox1*) is expressed in the first shell valve field of the aculiferan polyplacophorans *Acanthochitona fascicularis* and *Acanthochitona rubrolineata*, as well as in the spicule‐bearing cells of the aculiferan neomeniomorph *Wirenia argentea* (Figure 2A,B) (Fritsch et al. 2015, 2016; Huan et al. 2020). In these aculiferans, *lab* (*hox1*) is expressed in the anterior‐most region, adjacent to the prototroch. In contrast, in conchiferans, such as scaphopods, cephalopods, gastropods, and bivalves, the *lab* (*hox1*) expression domain encompasses the entire shell field rudiment(s) (Figure 2C–F) (Samadi and Steiner 2009; Fritsch et al. 2015, 2016; Wollesen et al. 2018; Huan et al. 2020; Salamanca‐Díaz et al. 2021; Barrera Grijalba et al. 2023). This difference suggests that the expression of *lab* (*hox1*) in aculiferans may represent an ancestral feature, where *lab* (*hox1*) is expressed in a staggered pattern along the anterior‐posterior axis, unlike the extended anterior‐posterior domain observed in conchiferans. In conchiferans, the expression domain may have expanded secondarily to include the posterior region surrounding the entire shell field.

**Figure 8:**
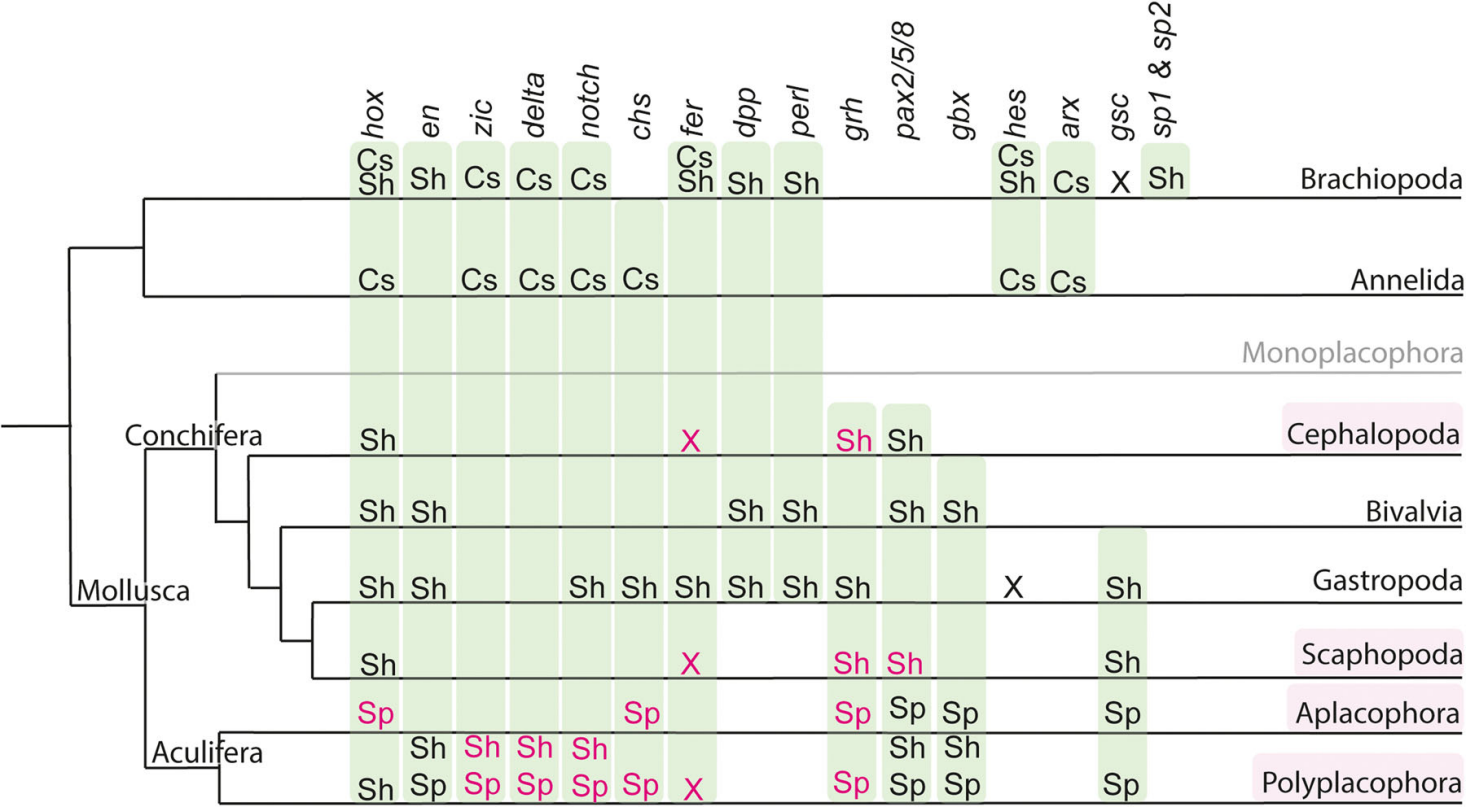
Comparative gene expression in lophotrochozoan shell fields, spicule‐bearing cells, and chaetoblasts. This cladogram highlights the expression of selected genes as revealed by in situ hybridization in the shell fields, spicule‐bearing cells, and chaetoblasts at various developmental stages of mollusks, brachiopods, and annelids. Branch lengths do not reflect phylogenetic distance or the relative evolutionary origin of taxa. Taxa and genes analyzed in this study are labeled in pink. Monoplacophora is shaded in gray, as no gene expression data are currently available. Cs, chaetal sacs; Sh, Shell; Sp, Spicules; X, gene expression was not revealed in developmental stages studied so far. Phylogeny adapted from Laumer et al. (2019) and Kocot et al. (2020). Information on gene expression: Gastropoda (Lartillot et al. 2002; O'Brien and Degnan 2003; Jackson et al. 2007; Hashimoto et al. 2012; Perry et al. 2015; Herlitize et al. 2018); Scaphopoda (Wollesen et al. 2018; present study); Bivalvia (Wollesen et al. 2015b, 2017); Cephalopoda (Wollesen et al. 2015b; present study); Polyplacophora (Wollesen et al. 2015b, 2017; Huan et al. 2020; present study); Aplacophora (Scherholz et al. 2017; Wollesen et al. 2017); Brachiopoda (Luo et al. 2015; Schiemann et al. 2017; Wernström et al. 2022); Annelida (Schiemann et al. 2017; Gazave et al. 2016).

Other genes expressed in the shell fields of certain molluscan clades, as well as in the spicule‐bearing cells of aculiferans, include *grh*, *gsc*, *gbx*, *chs*, *notch*, and *delta* (Figure 8; Supplementary Information Table 4) (Wollesen et al. 2017; present study). Orthologous genes expressed in the spicule‐bearing cells and cells giving rise to the shell plates of the polyplacophoran *A. fascicularis* argue for a shared evolutionary history of cell populations. This notion is also supported by similar biomineralization‐related matrix proteins present in the shell plates and spicules of the polyplacophoran *Acanthopleura loochooana* (Liu et al. 2024). Shared proteins comprise tyrosinase‐hemocyanin, carbonic anhydrases, von‐Willebrand‐factor type A, cadherin, and glycine‐rich unknown proteins (Liu et al. 2024). Previous studies have shown that *fer* is expressed in the gastropod shell field and prototroch (Jackson et al. 2007; Hashimoto et al. 2012). However, in the conchiferans *Antalis entalis* and *Xipholeptos notoides* and the polyplacophoran *Acanthochitona fascicularis*, *fer* has not been detected in the shell fields (Figure 8). Nevertheless, most mollusks with a prototroch studied so far express *fer* in this structure (present study; Hashimoto et al. 2012). This suggests that *fer* was likely not expressed in the shell fields or spicule‐bearing cells of the last common molluscan ancestor and was secondarily recruited for shell field formation in gastropods. Interestingly, in *A. fascicularis*, *fer* is expressed in the apical organ, which appears to be unique to polyplacophorans, as it has not been observed in other mollusks.

### 4.2 Spicules Are Deeply Rooted in Lophotrochozoan Evolutionary History

The variability in the structure, composition, and formation processes of spicules, chaetae, and setae among Lophotrochozoa complicates efforts to infer their evolutionary origins. In mollusks, both aplacophoran and polyplacophoran spicules are composed of calcium carbonate and are secreted by epidermal cells (Hoffman 1949; Haas 1979, 1981; Leise 1984, 1986; Scheltema 1985). In polyplacophorans, spicules are embedded in the cuticle of the girdle and secreted by individual or multiple invaginated cells (Haas 1979; Leise 1984; Kingsley et al. 2012). In the polyplacophoran *Mopalia mucosa*, spicules remain intracellular until the trochophore settles (Leise 1984). Polyplacophorans exhibit various types of spicules with differing anatomy, location, formation processes, and presumed functions (Leise 1984, 1986; Fischer et al. 1980). In the aplacophoran chaetodermomorphs and neomeniomorphs, spicules may be formed by one or more cells (Kingsley et al. 2012). Spicules can be plate‐like (Pruvot 1890), spindle‐like (Baba 1938), discoid, hollow, or solid (Pruvot 1890; reviewed in Kniprath 1981; Kingsley et al. 2012). In the deep‐sea neomeniomorph *Helicoradomenia* sp., spicules are secreted by a single cell, with surrounding support cells (Kingsley et al. 2012). The secretory cell forms a crystallization chamber lined with short microvilli in the apical region, as has also been described for other aplacophorans and polyplacophorans (Haas 1981). Our data show that despite differences in formation processes and anatomy, spicule‐bearing cells in polyplacophorans and neomeniomorphs share the expression of highly conserved genes such as *grh*, *gsc*, *gbx* as well as structural genes like *chs* (Supporting Information Table 4) (Figure 8; Wollesen et al. 2017). Additional genes expressed in the spicule‐bearing cells of polyplacophorans include *zic*, *delta*, and *notch* (Figure 8). Transcriptomic data indicate that the latter gene sequences are also present in neomeniomorphs, though riboprobes could not be synthesized (data not shown).

Elongated spicule‐like hard parts are also found in other lophotrochozoans, and histological techniques have revealed similarities between annelid and brachiopod chaetoblasts (Gustus and Cloney 1972; summarized in Schiemann et al. 2017). Both cell types exhibit long apical microvilli that project through a channel lined by support cells (Gustus and Cloney 1972). The secreted annelid and brachiopod chaetae are composed of protein and β‐chitin, although exceptions exist, such as calcified chaetae in euphrosine annelids (Müller et al. 2021). A recent study on the annelid *Capitella teleta* also showed that chaetae may be transient, appearing only during a short developmental window (Tilic et al. 2024). The spicule‐secreting cells of neomeniomorphs and the chaetoblasts of annelids and brachiopods are morphologically similar (cf. Kingsley et al. 2012; Schiemann et al. 2017). Both cell types have microvilli lining the basal (and inner lateral) parts of the cells, with longer microvilli in brachiopods and annelids and shorter ones in neomeniomorphs. It has also been demonstrated that the polyplacophoran *M. mucosa* possesses hair cells that secrete spicules (Leise and Cloney 1982). Previous studies have shown that *lab* (*hox1)*, *post2*, *arx, zic, notch, delta*, and *hes* genes are expressed in the chaetae of annelids and brachiopods (Figure 8; Schiemann et al. 2017; Supporting Information Table 4). Our study is the first to demonstrate that *notch, delta, lab (hox1)*, and *zic* are also expressed in the spicules of aculiferan representatives (Figure 8). The plasticity of spicule anatomy among aculiferans (even within groups like the neomeniomorphs) (Kingsley et al. 2012), along with the variability in annelid chaetae and the expression of deeply conserved transcription factors, suggests that the overall anatomy of aculiferan spicules and annelid and brachiopod chaetae may be homologous, though individual details in these hard structures could have evolved differently. Fossils like *Wiwaxia* show spicules with differing chitinous structures, while *Halkieria* has phosphate‐filled spicules (reviewed by Todt et al. 2008).

Additionally, we observed the expression of *chs* in the spicule‐bearing cells of polyplacophorans and neomeniomorphs. *Chs* is known to play a role in the formation of the conchiferan shell field (Weiss et al. 2006). In neomeniomorphs like *Wirenia argentea*, this finding aligns with expectations, as their cuticle contains chitin (Peters 1972; Furuhashi et al. 2009). While polyplacophoran trochophore larvae also possess a cuticle covering their shell, *chs* expression is notably restricted to spicule‐bearing cells, raising intriguing questions about why this expression is localized solely in these cells.

### 4.3 Shell Fields of Mollusks and Brachiopods —A Genetic Link Across Evolution?

Among Spiralia, shell fields are present in both Mollusca and Brachiopoda. Despite the aforementioned similarities between molluscan spicule‐bearing cells and brachiopod chaetoblasts, the shell fields of these two taxa differ significantly in anatomical, ontogenetic, and molecular genetic traits. While most brachiopod shells are composed of calcium carbonate, similar to those of mollusks, the shells of linguliform brachiopods consist of an organic matrix combined with calcium phosphate (apatite) minerals (Jackson et al. 2015; Santagata 2015). Brachiopods possess a dorsal and a ventral shell valve in contrast to bivalves with lateral shell valves. Other mollusks have shell fields primarily located on the dorsal side, which extend to the lateral sides during growth.

The molecular genetic mechanisms regulating brachiopod shell development exhibit both differences and similarities compared to those of mollusks. The Hox genes *lab* (*hox1*) and *antp* (*hox7*) are expressed in the periostracum, the shell‐forming epithelium of brachiopod larvae (Schiemann et al. 2017; Figure 8). *Lab* (*hox1*) is also consistently expressed in the shells of all molluscan representatives studied so far (see Figure 2 for details on *hox1* expression in mollusks), however, some aculiferan and conchiferan representatives express many additional Hox genes in their shell fields. For example, the scaphopod *Antalis entalis* expresses five Hox genes in its shell field, while the gastropod *Lottia goshimai* and polyplacophorans express all Hox genes in their shell fields (except *antp* (*hox7*) in gastropods and *post1* in polyplacophorans) (Supporting Information Table 4; Fritsch et al. 2015, 2016; Wollesen et al. 2018; Huan et al. 2020). This suggests that the last common molluscan ancestor likely expressed all Hox genes in its shell field(s).

Molluscan and brachiopod shell fields express other evolutionary highly conserved transcription factor encoding genes such as *engrailed*, in addition to other genes such as *dpp* (*bmp2/4*) and *perlucin* (Figure 8). *Chitin synthase* gene orthologs are also expressed in the brachiopod shell fields but their exact expression domain has not yet been documented via in situ hybridization (Luo et al. 2015). The role of *fer* in shell field formation, as shown for gastropods, could not be confirmed for aculiferans, nor for the conchiferan scaphopods and cephalopods. Instead, the last common molluscan ancestor probably expressed *fer* already in the prototroch and maybe the apical organ of its larva. A number of genes has been documented to be expressed in molluscan but not brachiopod shell fields, among them *pax2/5/8*, *gbx*, and *gsc*. Other genes such as *sp1*, *sp2*, or Hes genes have only been shown to be expressed in brachiopod shell fields (Figure 8).

Future studies, including single‐cell or nucleus sequencing approaches, will provide a more comprehensive understanding of which transcription factors are expressed in brachiopod and molluscan shell fields.

### 4.4 Outlook

This study demonstrates that the transcription factors *hox1*, *grainyhead*, *goosecoid*, *notch*, *delta*, and *zic*, as well as structural gene *chitin synthase* are expressed in the shell fields and/or spicule‐bearing cells of aculiferan and conchiferan mollusks. A role of *fer* in shell field formation as shown for gastropods could not be confirmed for aculiferans, nor for the conchiferan scaphopods and cephalopods. Instead, the last common molluscan ancestor probably expressed *fer* already in the prototroch of its larva. Our results show that some of the above‐mentioned candidate genes are up‐ and downregulated in the shell fields and/or the spicule‐bearing cells during distinct ontogenetic periods hinting toward a special role of these genes and asking for a better characterization of spiralian developmental stages.

## Author Contributions

Tim Wollesen designed the project, interpreted the data, compiled all figures and tables, and wrote the manuscript. Tim Wollesen and Sonia Victoria Rodríguez Monje raised developmental stages of *A. fascicularis, Xipholeptos notoides*, and *Antalis entalis*. Emanuel Redl and Maik Scherholz raised developmental stages of *W. argentea*. Gabriela Ariza Aranguren performed the phylogenetic analysis under supervision of Cristian Camillo Barrera Grijalba and Tim Wollesen. Tim Wollesen, Sonia Victoria Rodríguez Monje, Kathrin Lunzer, and Cristian Camillo Barrera Grijalba performed the cloning experiments and the synthesis of the riboprobes. In situ experiments were carried out by Tim Wollesen, Cristian Camillo Barrera Grijalba, Sonia Victoria Rodríguez Monje, and Kathrin Lunzer. All authors approved the final version of the manuscript.

## Conflicts of Interest

The authors declare no conflicts of interest.

## Supporting information

Supporting Material Information Shell field MS_240225.
