# Supplementary material for: Molluscan Shells, Spicules, and Gladii Are Evolutionarily Deeply Conserved
Source: J Exp Zool B Mol Dev Evol. 2025 Apr 9;344(4):198–213. doi: 10.1002/jez.b.23294 (PMC12046283; doi:10.1002/jez.b.23294)
Supplement: Supplementary file 1 — Supporting Material Information Shell field MS_240225. [file JEZ-344-198-s001.pdf]

Supplementary Material Information:

Molluscan shells, spicules, and gladii are evolutionarily deeply conserved

Cristian Camillo Barrera Grijalba[+], Sonia Victoria Rodríguez Monje[+], Gabriela Ariza Aranguren, Kathrin Lunzer, Maik Scherholz, Emanuel Redl, Tim Wollesen[*]

Department of Evolutionary Biology, Faculty of Life Sciences, University of Vienna, Djerassiplatz 1, 1030 Vienna, Austria.

[+]First co-authors

*Corresponding author: Tim Wollesen; Email: tim.wollesen@univie.ac.at

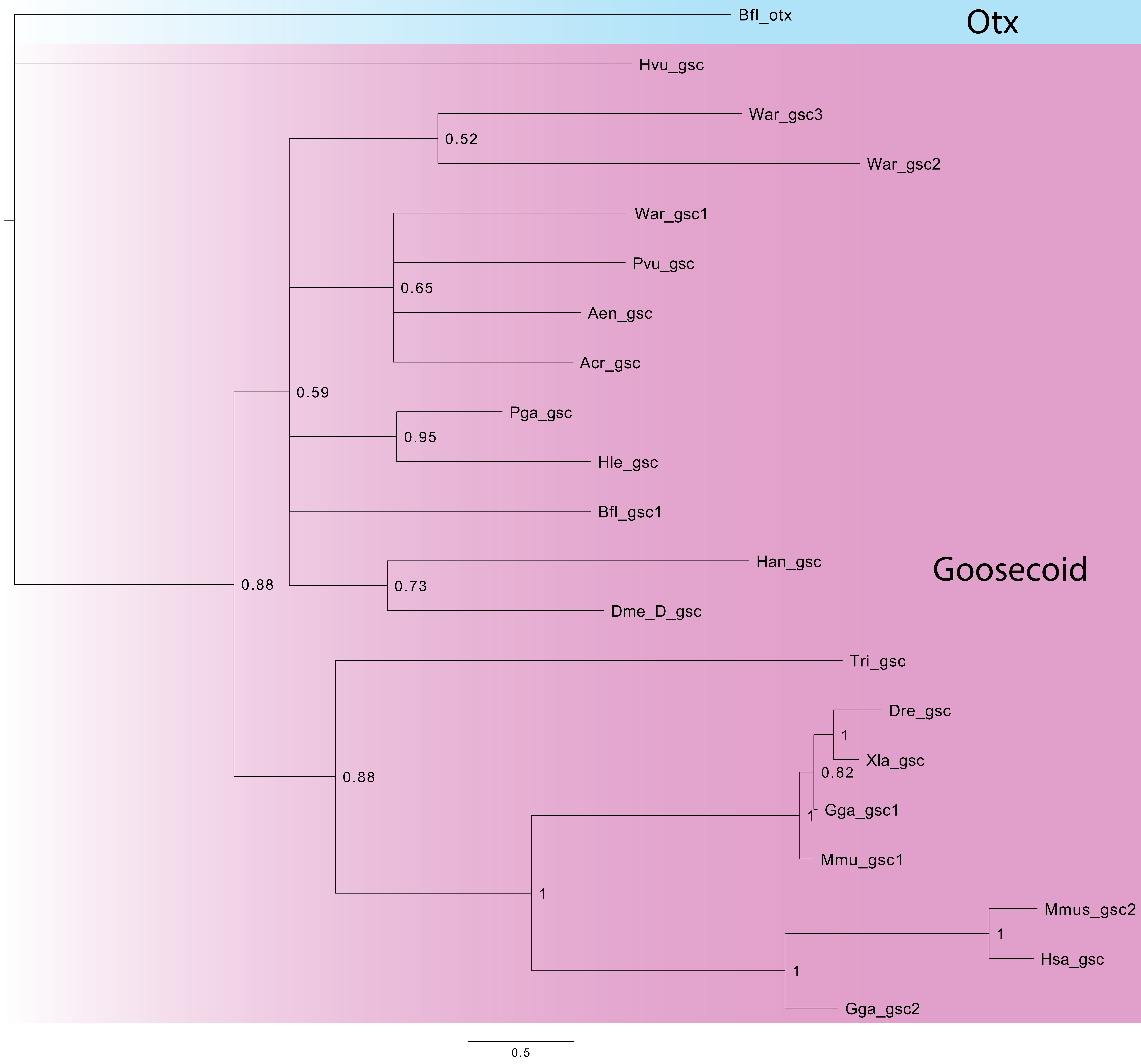
Otx
Bfl_otx
Hvu_gsc
War_gsc3
0.52
War_gsc2
War_gsc1
Pvu_gsc
0.65
Aen_gsc
Acr_gsc
0.59
Pga_gsc
0.95
Hle_gsc
Bfl_gsc1
Han_gsc
Goosecoid
0.88
0.73
Dme_D_gsc
Tri_gsc
Dre_gsc
1
Xla_gsc
0.88
0.82
Gga_gsc1
1
Mmu_gsc1
1
Mmus_gsc2
1
Hsa_gsc
1
Gga_gsc2
0.5

Figure 1. Orthology analysis of *gsc* amino acid sequences from representatives across Metazoa. The genes for *Acanthochitona fascicularis (*Acfa*), Antalis entails* (Aen), and *Wirenia argentea (*War*)* are highlighted. The outgroup is the *Otx* transcription factor subfamily. Bootstrap values associated with each relationship are shown. For abbreviations and references consult tables 1, 2 in supplementary data.

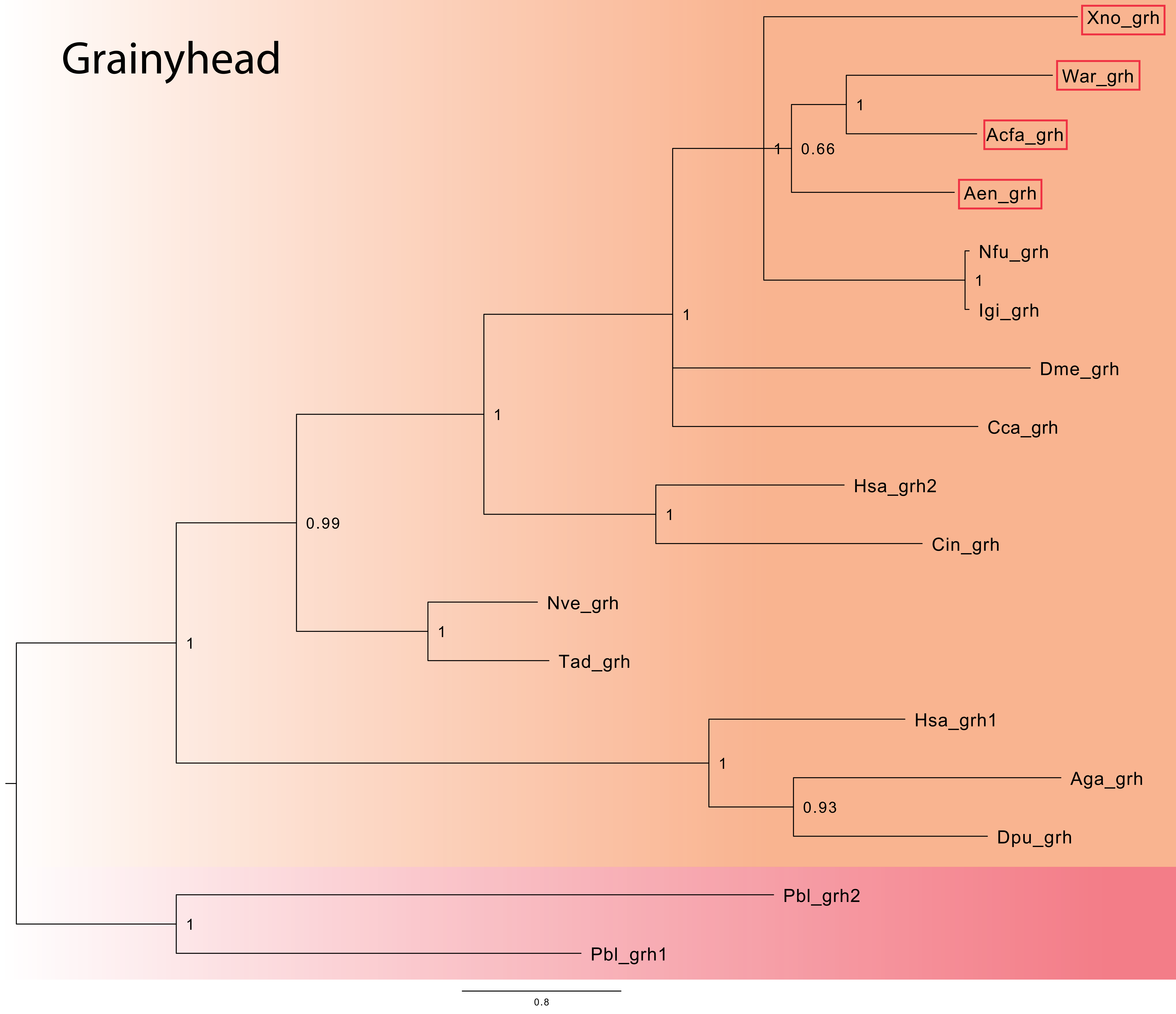
Grainyhead
Xno_grh
War_grh
1
Acfa_grh
1
0.66
Aen_grh
Nfu_grh
1
Igi_grh
1
Dme_grh
1
Cca_grh
Hsa_grh2
1
Cin_grh
0.99
Nve_grh
1
Tad_grh
1
Hsa_grh1
1
Aga_grh
0.93
Dpu_grh
Pbl_grh2
1
Pbl_grh1
0.8

Figure 2. Orthology analysis of *grh* amino acid sequences from metazoan representatives. The genes for *Acanthochitona fascicularis (*Acfa*), Antalis entails* (Aen), *Xipholeptos notoides* (Xno), and *Wirenia argentea (*War*)* are highlighted. The *grh* gene from *Phycomyces blakesleeanus* (Pba) was used as outgroup. For abbreviations and references see tables 1, 2 in supplementary data.

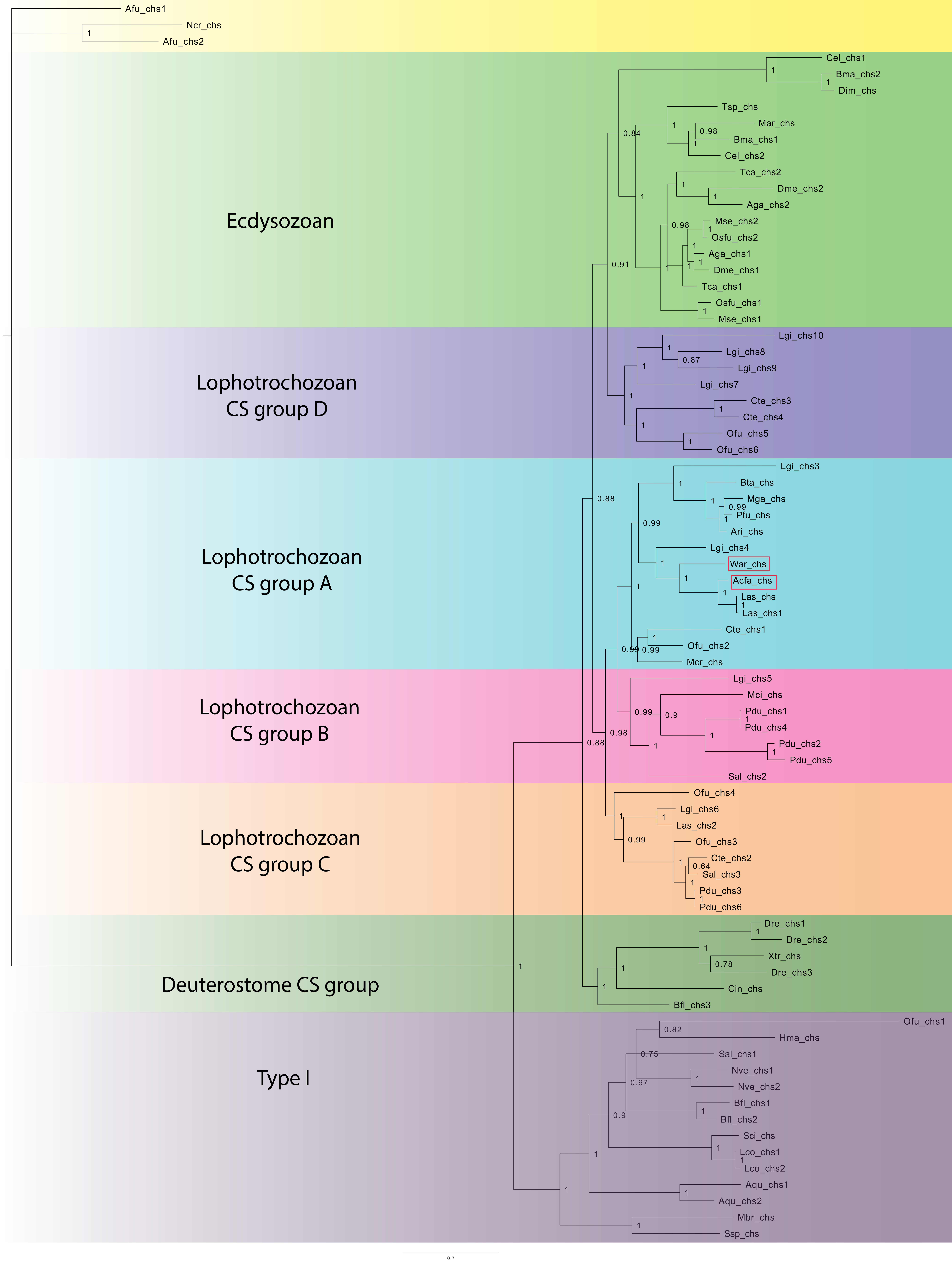

Afu_chs1
Ncr_chs
Afu_chs2
Ecdysozoan
Cel_chs1
Bma_chs2
Dim_chs
Tsp_chs
Mar_chs
Bma_chs1
Cel_chs2
Tca_chs2
Dme_chs2
Aga_chs2
Mse_chs2
Osfu_chs2
Aga_chs1
Dme_chs1
Tca_chs1
Osfu_chs1
Mse_chs1
Lophotrochozoan CS group D
Lgi_chs10
Lgi_chs8
Lgi_chs9
Lgi_chs7
Cte_chs3
Cte_chs4
Ofu_chs5
Ofu_chs6
Lophotrochozoan CS group A
Lgi_chs3
Bta_chs
Mga_chs
Pfu_chs
Ari_chs
Lgi_chs4
War_chs
Acfa_chs
Las_chs
Las_chs1
Cte_chs1
Ofu_chs2
Mcr_chs
Lophotrochozoan CS group B
Lgi_chs5
Mci_chs
Pdu_chs1
Pdu_chs4
Pdu_chs2
Pdu_chs5
Sal_chs2
Lophotrochozoan CS group C
Ofu_chs4
Lgi_chs6
Las_chs2
Ofu_chs3
Cte_chs2
Sal_chs3
Pdu_chs3
Pdu_chs6
Deuterostome CS group
Dre_chs1
Dre_chs2
Xtr_chs
Dre_chs3
Cin_chs
Bfl_chs3
Type I
Ofu_chs1
Hma_chs
Sal_chs1
Nve_chs1
Nve_chs2
Bfl_chs1
Bfl_chs2
Sci_chs
Lco_chs1
Lco_chs2
Aqu_chs1
Aqu_chs2
Mbr_chs
Ssp_chs
0.7

Figure 3. Orthology analysisof *chitin-synthase(chs)* amino acid sequences with bootstrap values for all the relationships. The genes for *Acanthochitona fascicularis* (Acfa) and *Wirenia argentea* (War) are highlighted. The *chitin-synthase* gene from fungi organisms was used as outgroup. For abbreviations and references see tables 1, 2 in supplementary data.

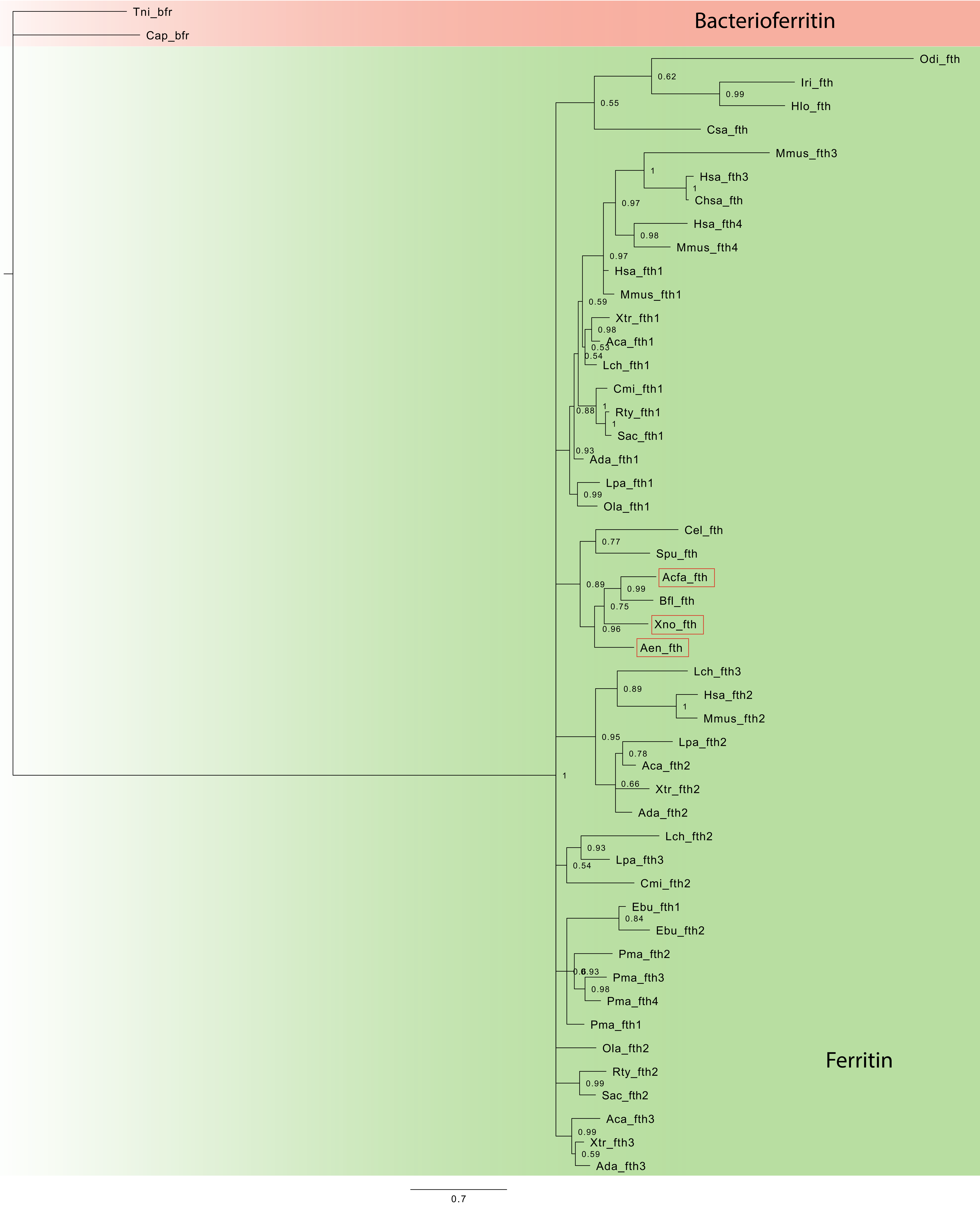

Bacterioferritin
Tni_bfr
Cap_bfr
Odi_fth
Iri_fth
Hlo_fth
Csa_fth
Mmus_fth3
Hsa_fth3
Chsa_fth
Hsa_fth4
Mmus_fth4
Hsa_fth1
Mmus_fth1
Xtr_fth1
Aca_fth1
Lch_fth1
Cmi_fth1
Rty_fth1
Sac_fth1
Ada_fth1
Lpa_fth1
Ola_fth1
Cel_fth
Spu_fth
Acfa_fth
Bfl_fth
Xno_fth
Aen_fth
Lch_fth3
Hsa_fth2
Mmus_fth2
Lpa_fth2
Aca_fth2
Xtr_fth2
Ada_fth2
Lch_fth2
Lpa_fth3
Cmi_fth2
Ebu_fth1
Ebu_fth2
Pma_fth2
Pma_fth3
Pma_fth4
Pma_fth1
Ola_fth2
Rty_fth2
Sac_fth2
Aca_fth3
Xtr_fth3
Ada_fth3
Ferritin
0.7

Figure 4. Orthology tree on *ferritin (fer/fth)* amino acid sequences compromising members of Metazoa. The genes for *Acanthochitona fascicularis* (Acfa)*, Antalis entails* (Aen), *Xipholeptos notoides* (Xno)*,* and *Wirenia argentea* (War) are highlighted. The gene *Bacterioferritin* (Bfr) was used as outgroup. For abbreviations and references see tables 1, 2 in supplementary data.

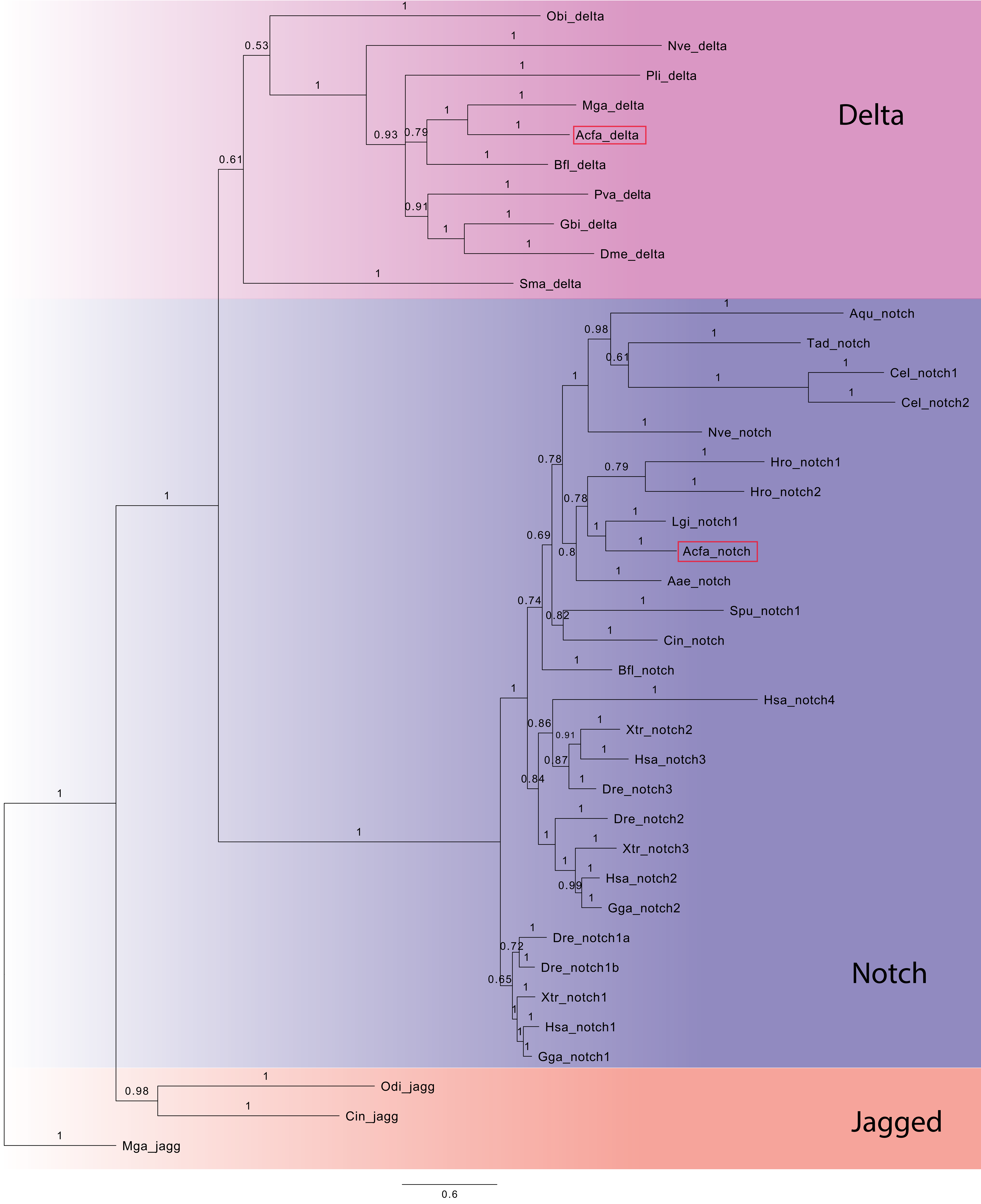

Delta
Notch
Jagged
Obi_delta
Nve_delta
Pli_delta
Mga_delta
Acfa_delta
Bfl_delta
Pva_delta
Gbi_delta
Dme_delta
Sma_delta
Aqu_notch
Tad_notch
Cel_notch1
Cel_notch2
Nve_notch
Hro_notch1
Hro_notch2
Lgi_notch1
Acfa_notch
Aae_notch
Spu_notch1
Cin_notch
Bfl_notch
Hsa_notch4
Xtr_notch2
Hsa_notch3
Dre_notch3
Dre_notch2
Xtr_notch3
Hsa_notch2
Gga_notch2
Dre_notch1a
Dre_notch1b
Xtr_notch1
Hsa_notch1
Gga_notch1
Odi_jagg
Cin_jagg
Mga_jagg
0.6

Figure 5. Orthology tree on *zic* gene including amino acid sequences across different taxa. The genes for *Acanthochitona fascicularis* (Acfa) and *Wirenia argentea* (War) are highlighted. The Zinc-finger antiviral protein (Zap) subfamily from yeast, was used as outgroup. For abbreviations and references see tables 1, 2 in supplementary data.

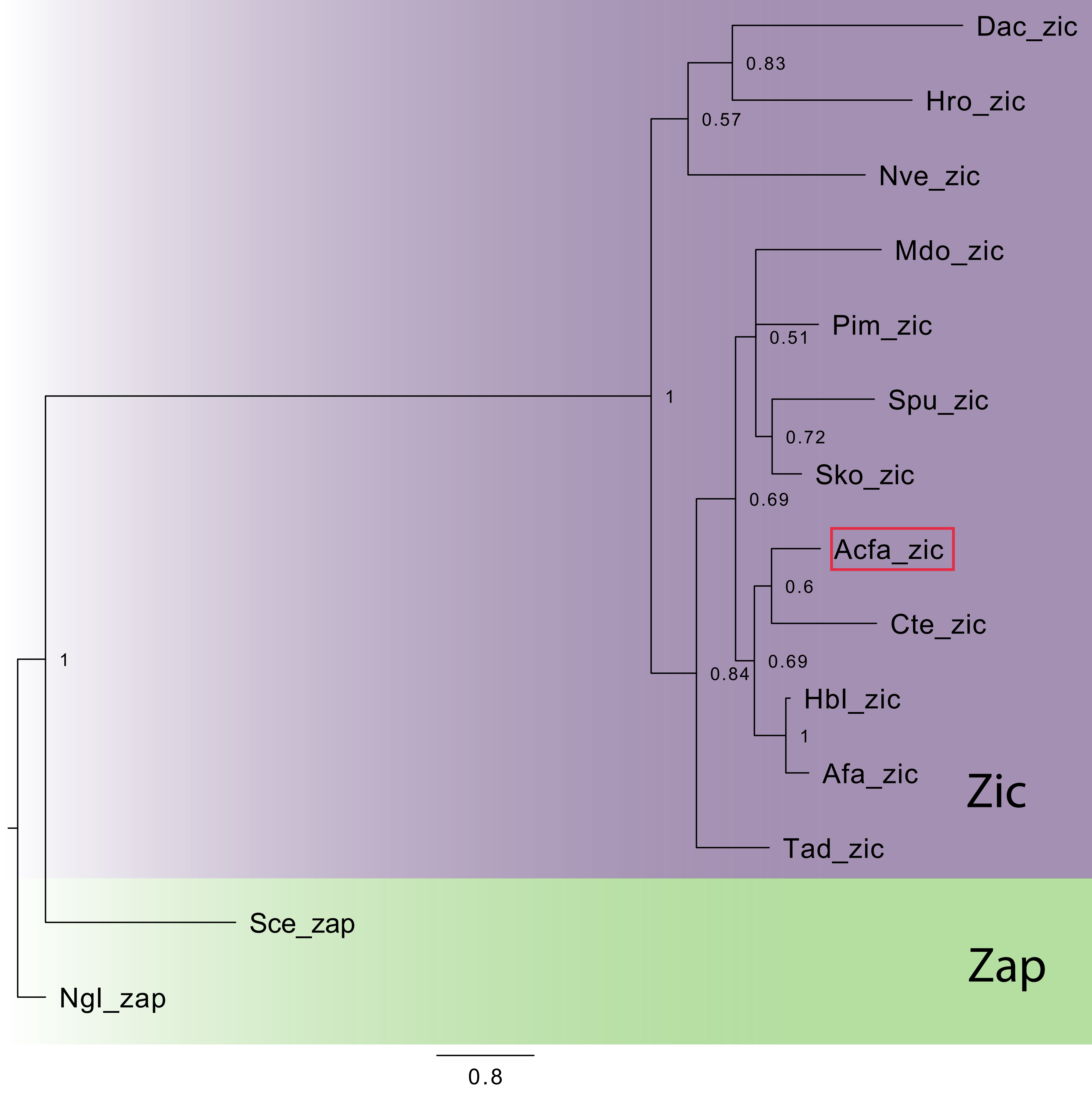

Dac_zic
0.83
Hro_zic
0.57
Nve_zic
Mdo_zic
Pim_zic
0.51
1
Spu_zic
0.72
Sko_zic
0.69
Acfa_zic
0.6
Cte_zic
1
0.69
0.84
Hbl_zic
1
Afa_zic
Zic
Tad_zic
Sce_zap
Zap
Ngl_zap
0.8

Figure 6. Orthology analysis on *notch* and *delta* amino acid sequences. Bootstrap values are indicated for all the relationships. The genes for *Acanthochitona fascicularis* (Acfa) and *Wirenia argentea* (War) are highlighted. The gene for the jagged1 protein was used as outgroup. For abbreviations and references see tables 1, 2 in supplementary data.

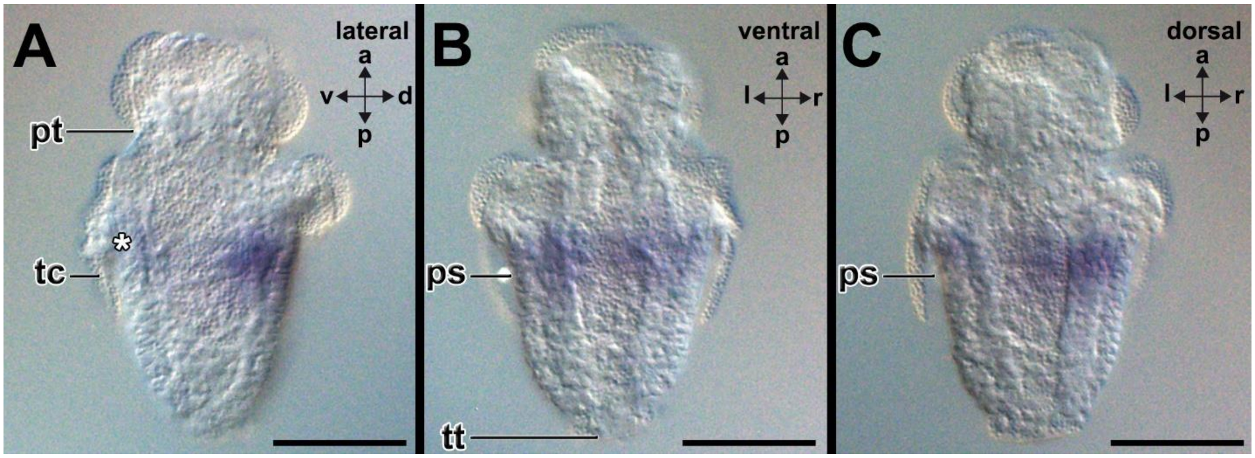

Figure 7 Expression of *hox1* in the late-stage larva (16 dph) of the neomeniomorph *Wirenia argentea.* Dorsal (d)-ventral (v), anterior (a)-posterior (p), and left (l)-right (r) axes indicate the orientation. Asterisks mark the mouth opening. **A-C:** *Hox1* is expressed by the spicule-bearing cells that line the tissue surrounding the peri-imaginal space (ps). Abbreviations: pt, prototroch; tc, test cell; tt, telotroch. Scale bars: 50 µm.

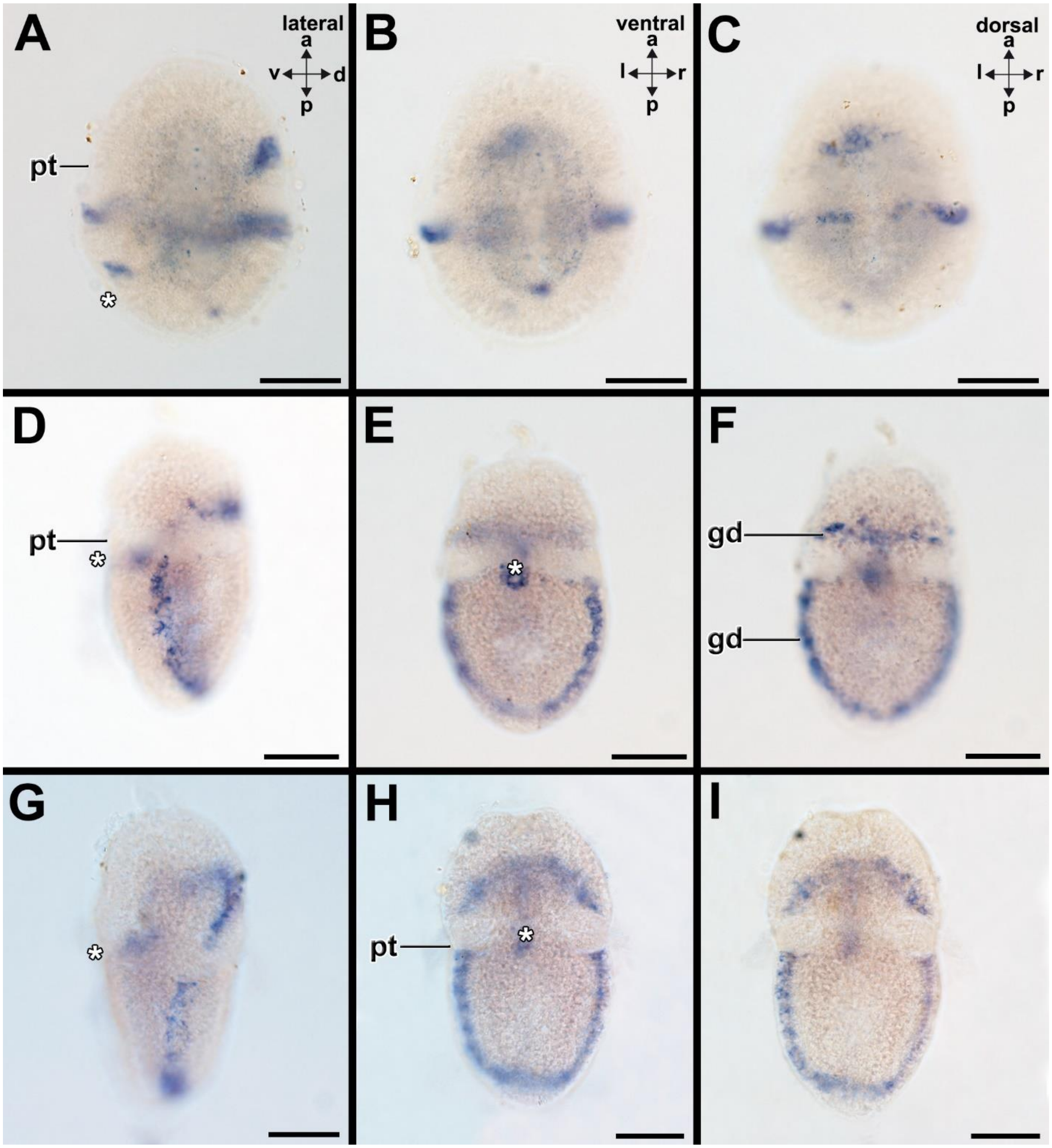

Figure 8 Expression of *goosecoid* in the polyplacophoran *Acanthochitona fascicularis.* Dorsal (d)-ventral (v), anterior (a)-posterior (p), and left (l)-right (r) axes indicate the orientation. Asterisks mark the mouth opening. **A-C:** Early trochophore larvae express *gsc* in the spicule-bearing cells of the forming girdle which is divided by the prototroch (pt). The spicule-bearing cells in the hyposphere are distributed from the lateral sides to via the dorsal side and form almost a continuous circle around the trochophore

larva. *Gsc* is also expressed anterior to the mouth opening and in a small domain in the posterior dorsal region that may correspond to the posterior-most girdle. **D-F:** Mid-stage trochophore larvae express *gsc* in the spicule-bearing cells of the girdle (gd) and around the mouth-opening. In these developmental stages, the *gsc+* spicule-bearing cells form a continuous band except the region of the prototroch. **G-I:** *Gsc*-expression of late-stage trochophore larvae resembles the condition found in mid-stage larvae. Scale bars: A-C: 50 µm

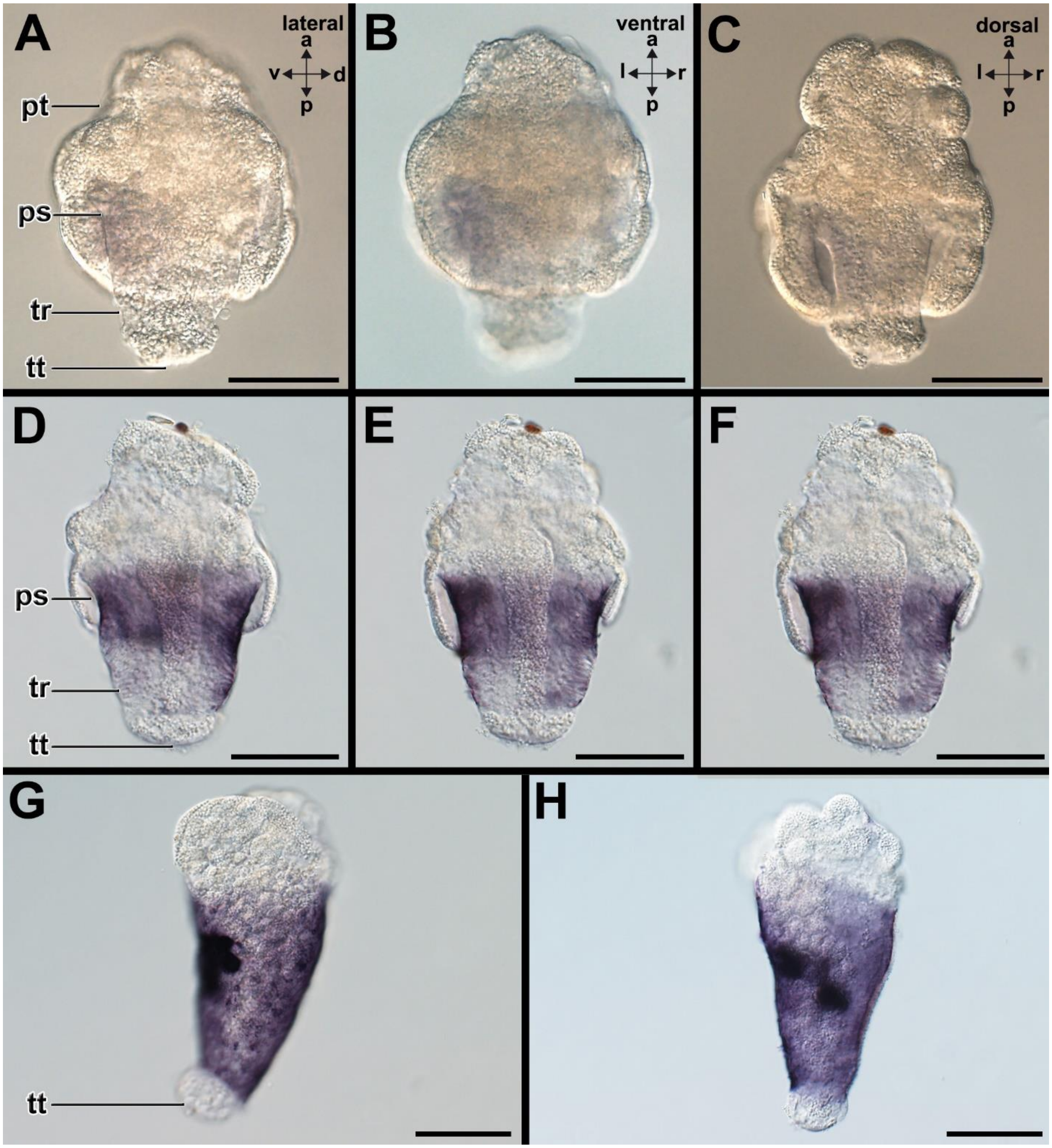

Figure 9 Expression of *goosecoid1* in the neomeniomorph *Wirenia argentea.* Dorsal (d)-ventral (v), anterior (a)-posterior (p), and left (l)-right (r) axes indicate the orientation. **A-C:** In mid-stage test cell larva (6-7dph) *gsc1* is expressed in the spicule-bearing cells that line the tissue surrounding the peri-imaginal space (ps) and the outgrowing trunk (tr). **D-F:** In late-stage test cell larva (16dph) *gsc1* is expressed in the spicule-bearing cells that line the tissue surrounding the peri-imaginal space and the

outgrowing trunk. The posterior-most region of the trunk is not *gsc1+*. **G-H:** Late-stage larvae (18dph) with *gsc1* expression in the trunk region. Abbreviations: pt, prototroch; tt, telotroch. Scale bars: 50 µm

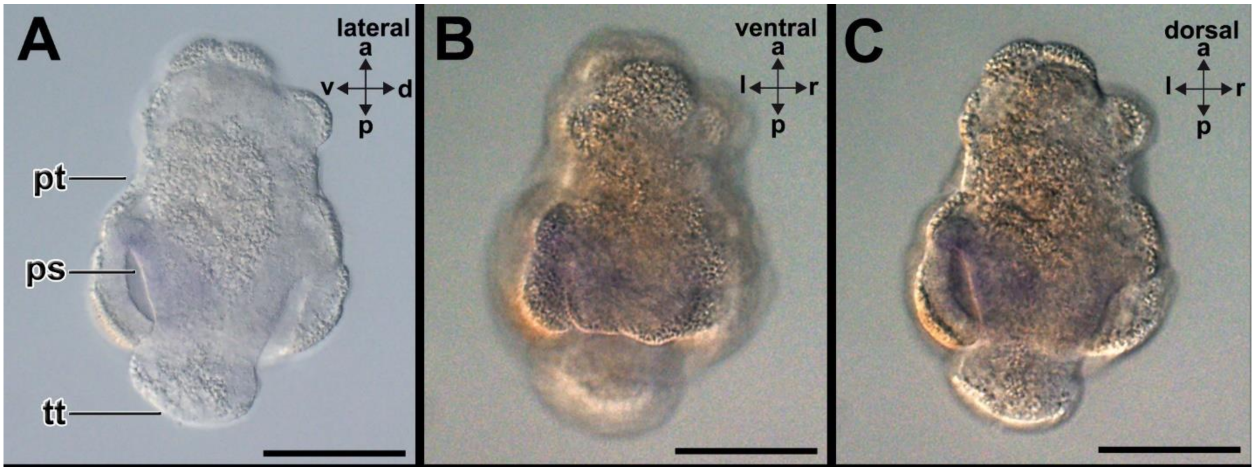

Figure 10 Expression of *goosecoid2 in* the neomeniomorph *Wirenia argentea.* Dorsal (d)-ventral (v), anterior (a)-posterior (p), and left (l)-right (r) axes indicate the orientation. **A-C:** In mid-stage test cell larva (6-7dph) *gsc2* is expressed in the spicule-bearing cells that line the tissue surrounding the peri-imaginal space (ps) and the outgrowing trunk. Individuals of other developmental stages could not be studied due to the lack of samples. Abbreviations: pt, prototroch; tt, telotroch. Scale bars: 50 µm

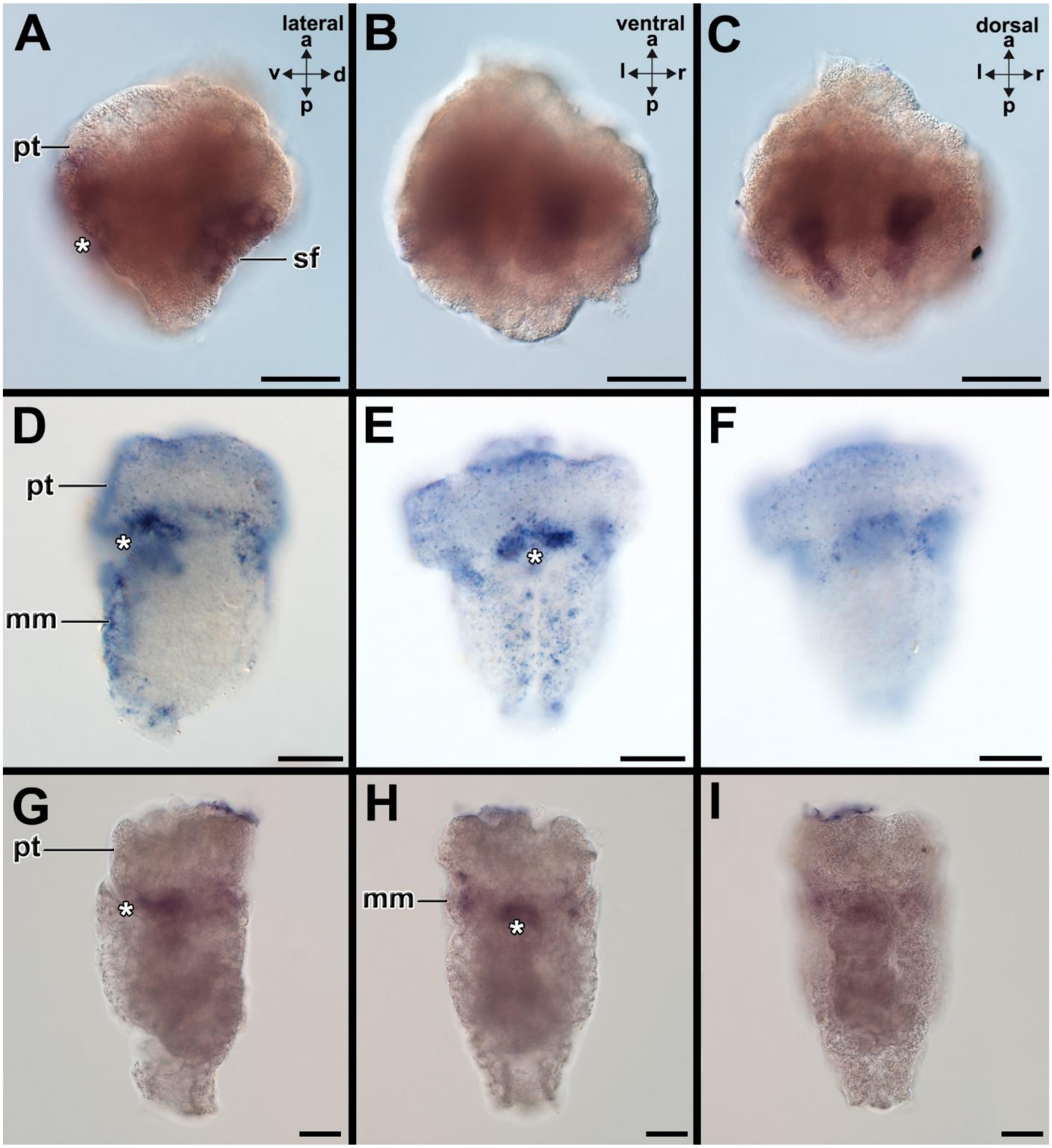

Figure 11 Expression of *goosecoid* in the scaphopod *Antalis entails.* Dorsal (d)-ventral (v), anterior (a)-posterior (p), and left (l)-right (r) axes indicate the orientation. Asterisks mark the mouth opening. **A-C:** *Gsc* is expressed in a paired domain in the forming shell field (sf) and in the region around the mouth of early-stage trochophores. **D-F:** Mid-stage trochophores express *gsc* along the entire mantle margin (mm)

and in the region surrounding the mouth. **G-I:** Late mid-stage trochophores express *gsc* around the mouth and along the anterior mantle margin. Abbreviations: pt, prototroch. Scale bars: 50 µm

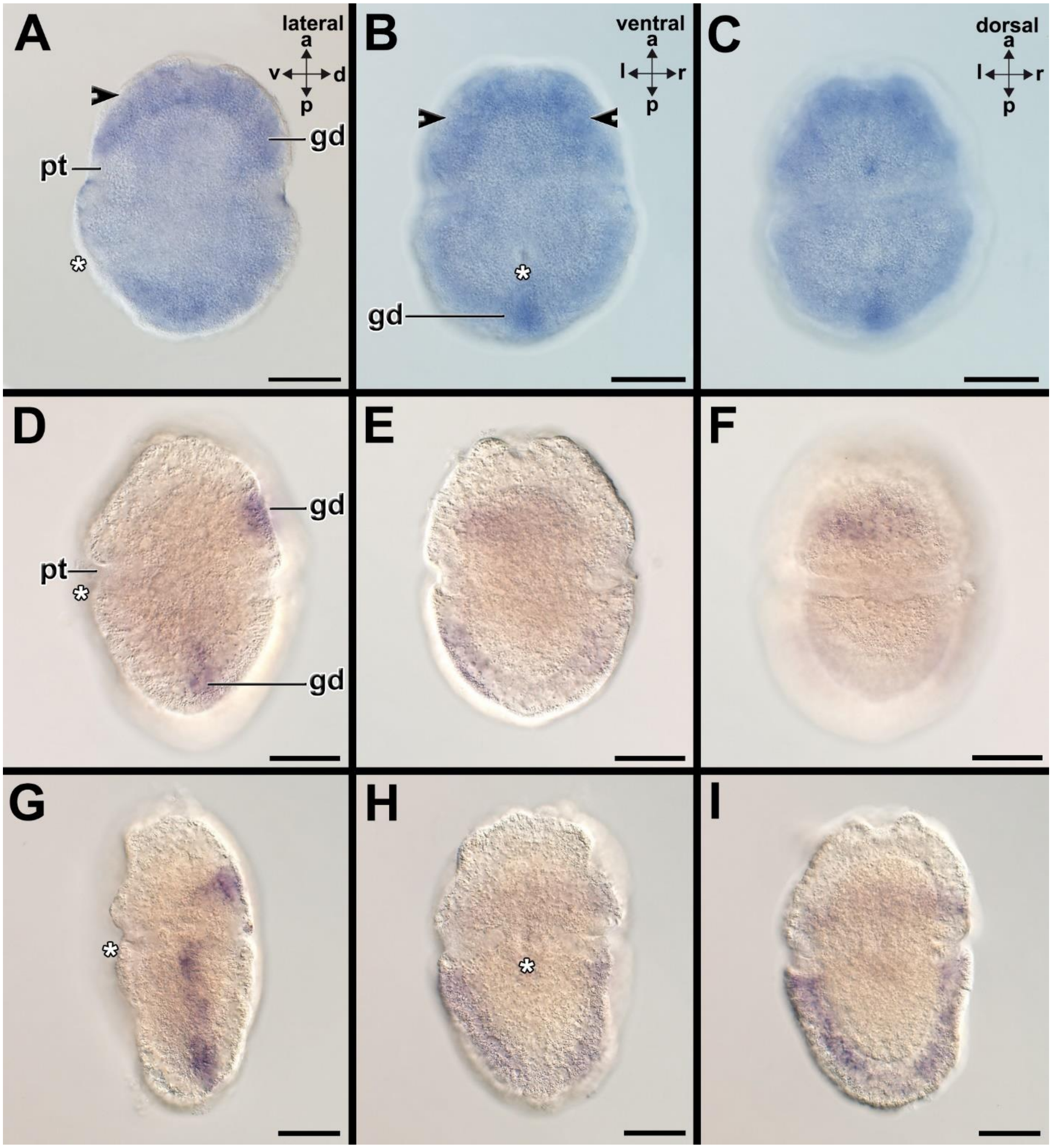

Figure 12 Expression of *grainyhead* in the polyplacophoran *Acanthochitona fascicularis.* Dorsal (d)-ventral (v), anterior (a)-posterior (p), and left (l)-right (r) axes indicate the orientation. Asterisks mark the mouth opening. **A-C:** Early trochophore larvae of the polyplacophoran *A. fascicularis* express *grh* in the posterior-most region of the forming girdle (gd), in addition to a domain anterior to the prototroch (pt) where the anterior girdle forms. Weak expression is also visible in epidermal cells of the lateral episphere

(arrowheads). **D-F:** Mid-stage trochophore larvae express *grh* in the spicule-bearing cells of the anterior and lateral girdle. The posterior-most domain of the girdle only houses few *grh*-expressing cells. **G-I:** Late-stage trochophore larvae express *grh* in the spicule-bearing cells of the anterior and lateral girdle. The posterior-most domain of the girdle only houses few *grh*-expressing cells. Scale bars: 50 µm

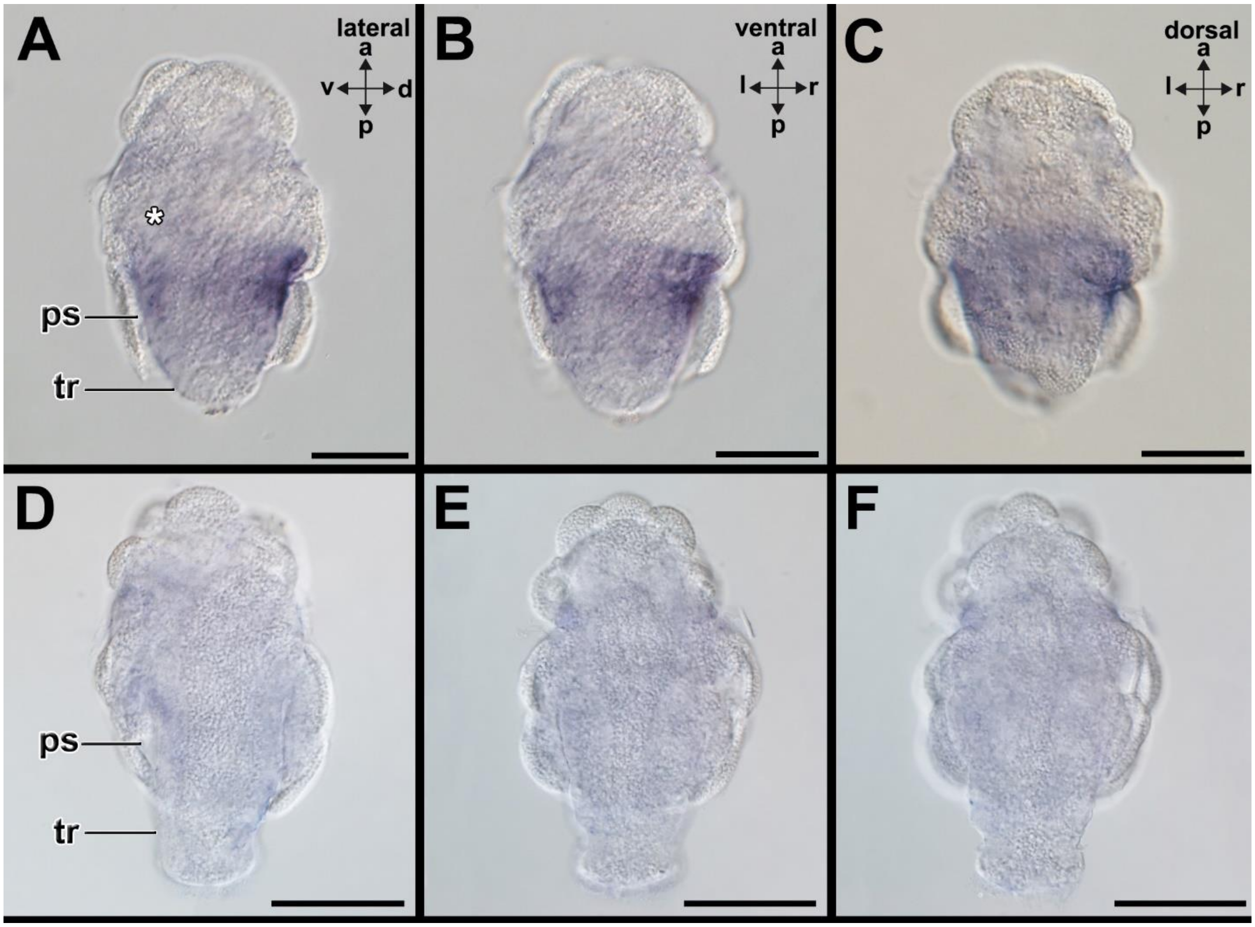

Figure 13 Expression of *grainyhead* in the neomeniomorph *Wirenia argentea*. Dorsal (d)-ventral (v), anterior (a)-posterior (p), and left (l)-right (r) axes indicate the orientation. Asterisks mark the mouth opening. **A-C:** In early-stage test cell larvae of the neomeniomorph *Wirenia argentea* (6-7dph) *grh* is expressed in the spicule-bearing cells that line the tissue surrounding the peri-imaginal space (ps) and the outgrowing trunk (tr). **D-F:** Subsequent developmental stages only faintly express *grh* in that region. Scale bars: 50 µm

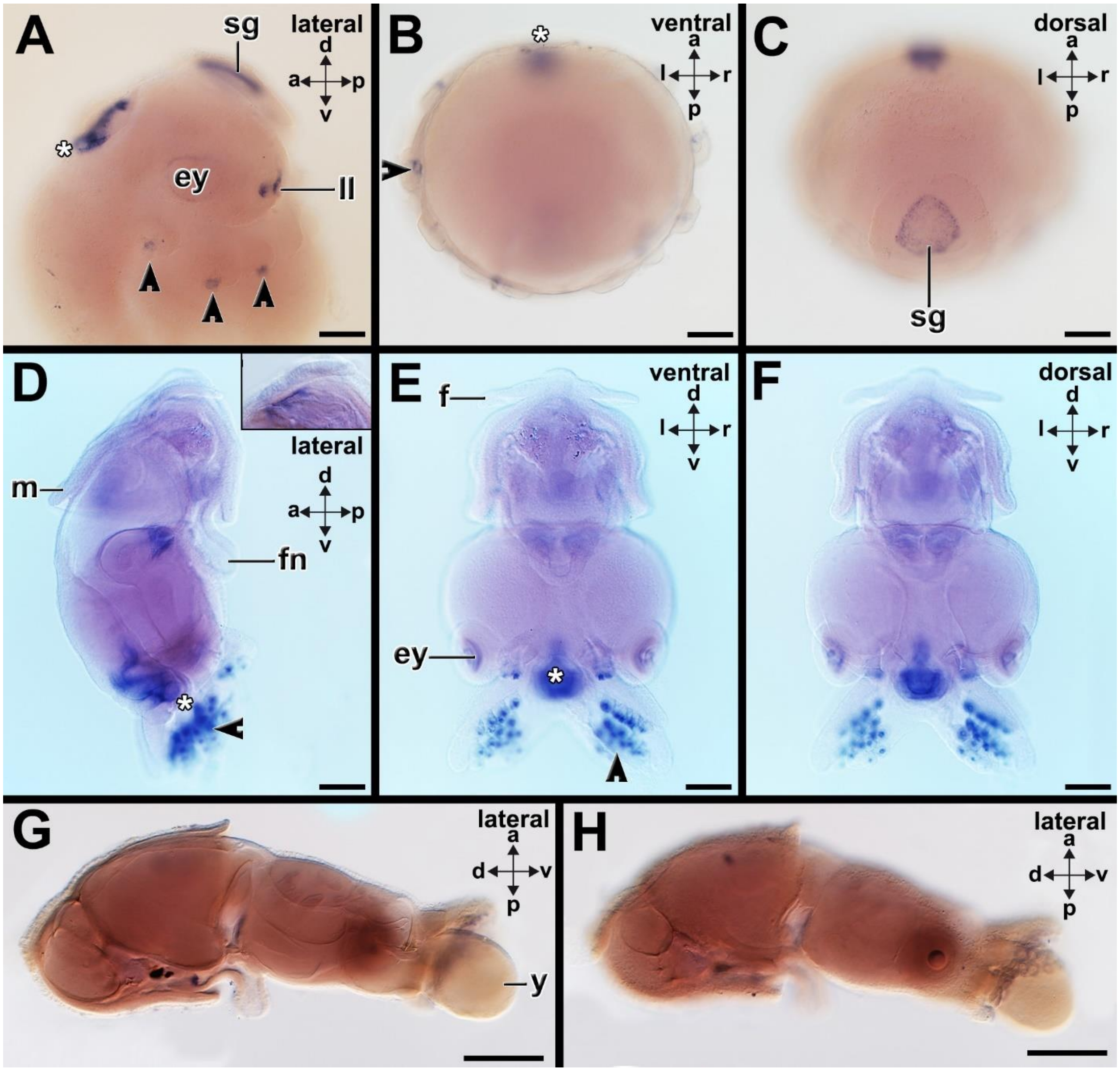

Figure 14 Expression of *grainyhead* in the cephalopod *Xipholeptos notoides.* Dorsal (d)-ventral (v), anterior (a)-posterior (p), and left (l)-right (r) axes indicate the orientation. Asterisks mark the mouth opening. **A-C:** In the early developmental stages (stage 19) of the cephalopod *Xipholeptos notoides grh* is expressed in the region of the shell gland (sg), the median regions of the developing arms, where the suckers develop (arrowheads), the mouth region (asterisk) where the jaw develops, and the region of the lateral lips (ll) posterior to the eyes. Slight expression is also visible in developing eyes (ey). **D-F:** More advanced developmental stages (stage 25) express *grh* in the suckers of the arms (arrowheads), the region

of the mouth with the jaw and the adjacent esophagus. Additional staining is visible in the developing eyes. **G-I:** No *grh*-expression was detected in subsequent developmental stages (stage 28). Abbreviations: f, fin; fn, funnel; m, mantle; y, yolk. Scale bars: 100 µm

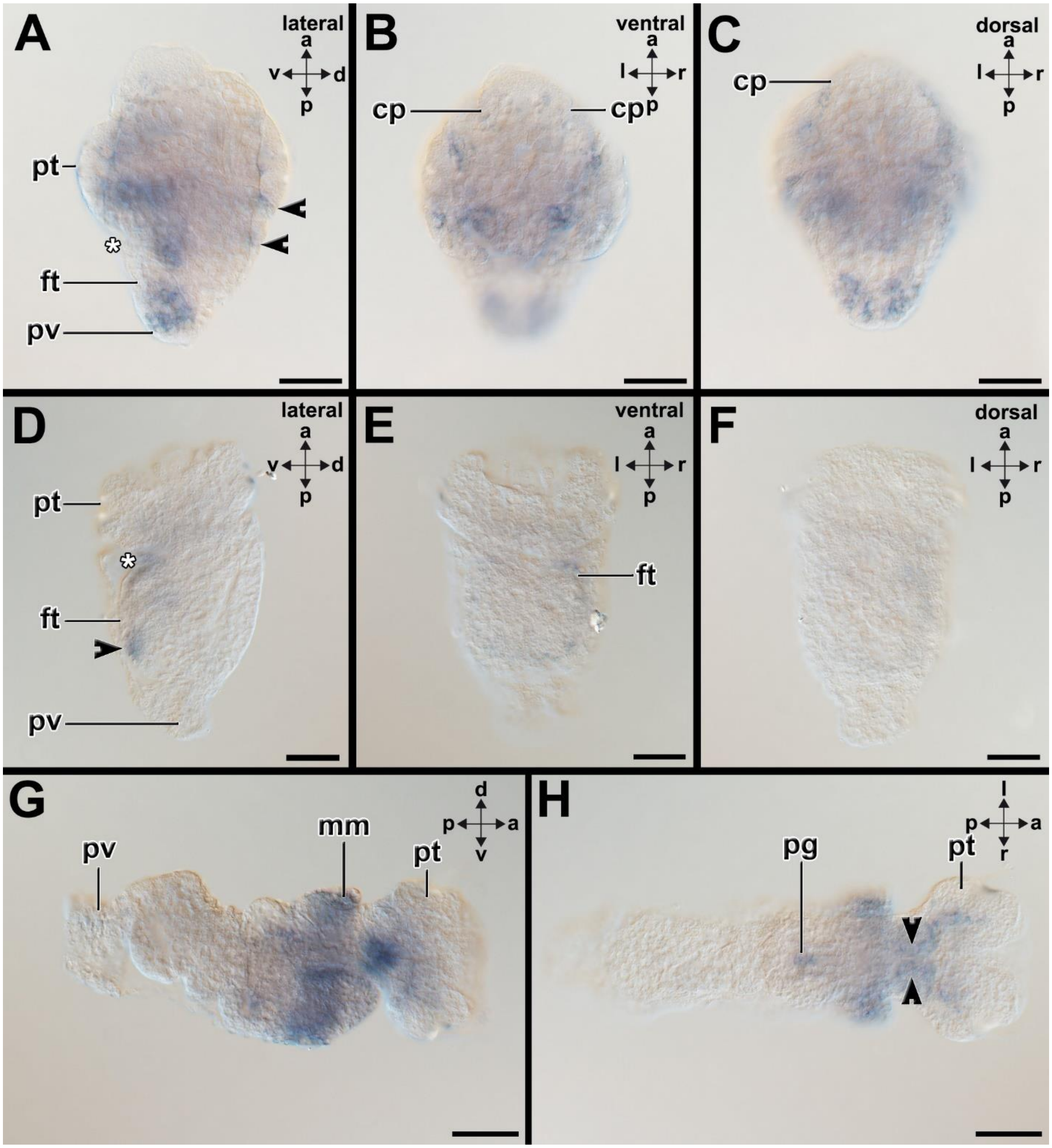

Figure 15 Expression of *grainyhead* in the scaphopod *Antalis entalis*. Dorsal (d)-ventral (v), anterior (a)-posterior (p), and left (l)-right (r) axes indicate the orientation. Asterisks mark the mouth opening. **A-C:** In early trochophore larvae of the scaphopod *Antalis entalis grh* is expressed in few cells of the forming shell field (arrowheads), the region of the forming pavilion (pv) and the lateral anterior foot (ft)

and mouth. *Grh*+ cells are also present in a region posterior to the cerebral pits (cp). **D-F:** In mid-stage trochophore *grh* is expressed in few cells surrounding the foot and in the region of the forming pedal ganglia (arrowhead). **G-H:** Late-stage trochophores express *grh* in their anterior mantle margin (mm), the region of the cerebral ganglia and/ or the apical organ (arrowheads), the pedal ganglia (pg) and the prototroch that is in the process of degeneration. Abbreviation: pt, prototroch. Scale bars: 50 µm

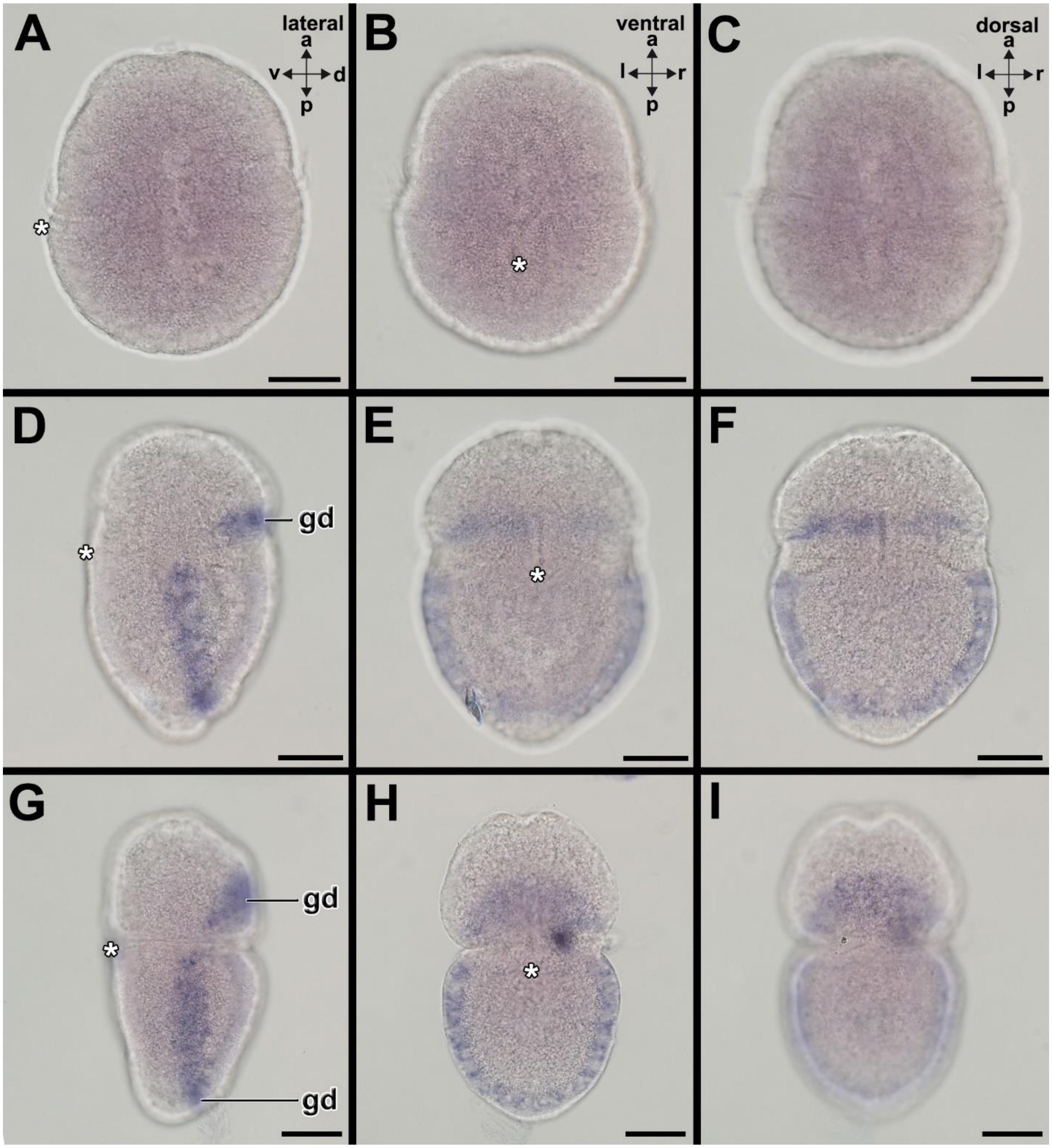

Figure 16 Expression of *chitin synthase* in the polyplacophoran *Acanthochitona fascicularis.* Dorsal (d)-ventral (v), anterior (a)-posterior (p), and left (l)-right (r) axes indicate the orientation. Asterisks mark the mouth opening. **A-C:** No *chs*-expression was observed in the early trochophore larva of the polyplacophoran *A. fascicularis*. **D-F:** Mid-stage larvae express *chs* in the spicule-bearing cells of the

girdle (gd). **G-I:** Late-stage trochophore larvae express *chs* in the spicule-bearing cells of the girdle. Scale bars: 50 µm

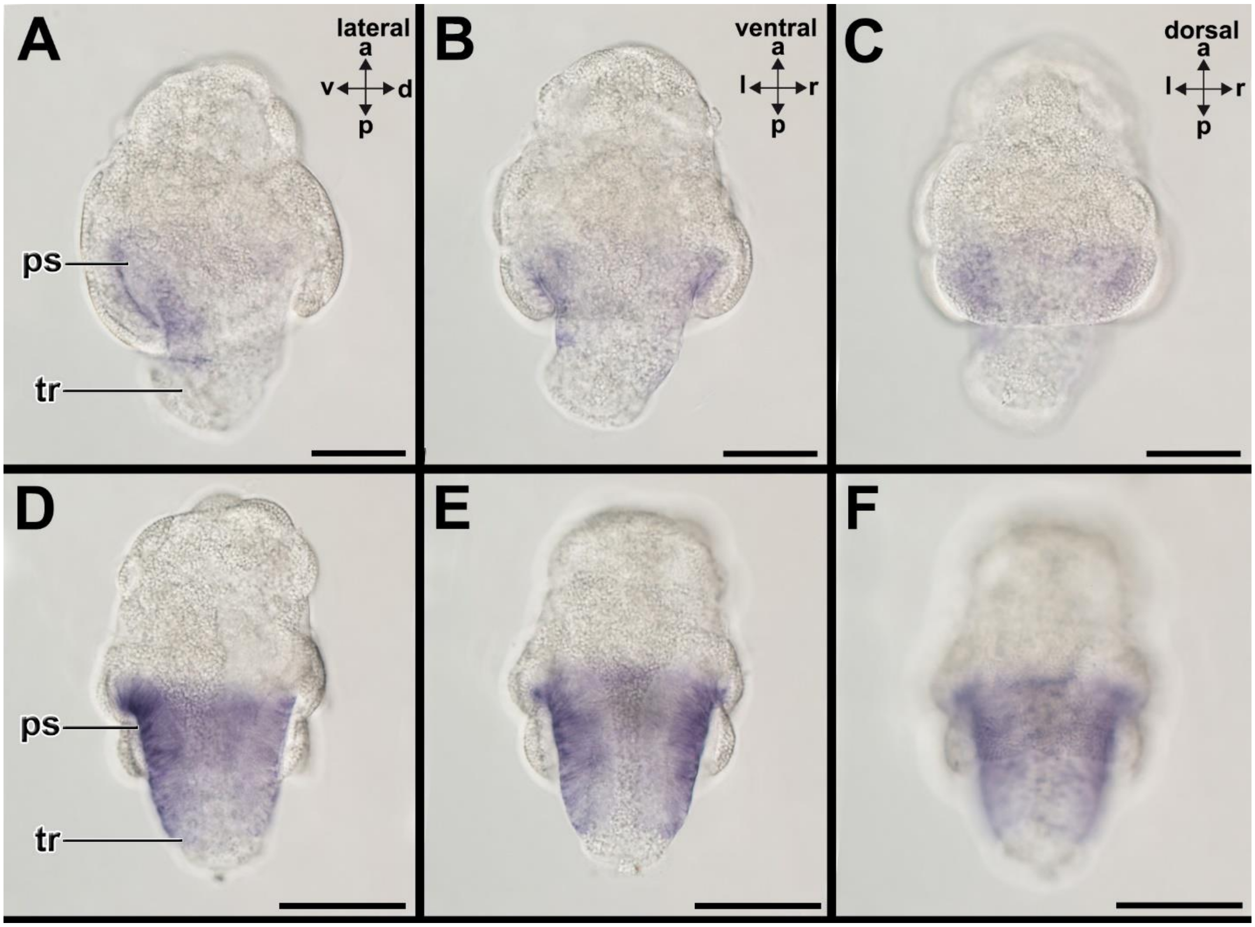

Figure 17 Expression of *chitin synthase* the neomeniomorph *Wirenia argentea.* Dorsal (d)-ventral (v), anterior (a)-posterior (p), and left (l)-right (r) axes indicate the orientation. **A-C:** In mid-stage test cell larvae (6-7dph) of the neomeniomorph *Wirenia argentea*, *chs* is expressed in the spicule-bearing cells that line the tissue of the outgrowing trunk (tr) surrounding the peri-imaginal space (ps). **D-F:** In late-stage larvae (16hph) *chs*+ cells are lining the epidermis of the trunk. The posterior-most region of the trunk does not exhibit *chs*+ cells. Scale bars: 50 µm

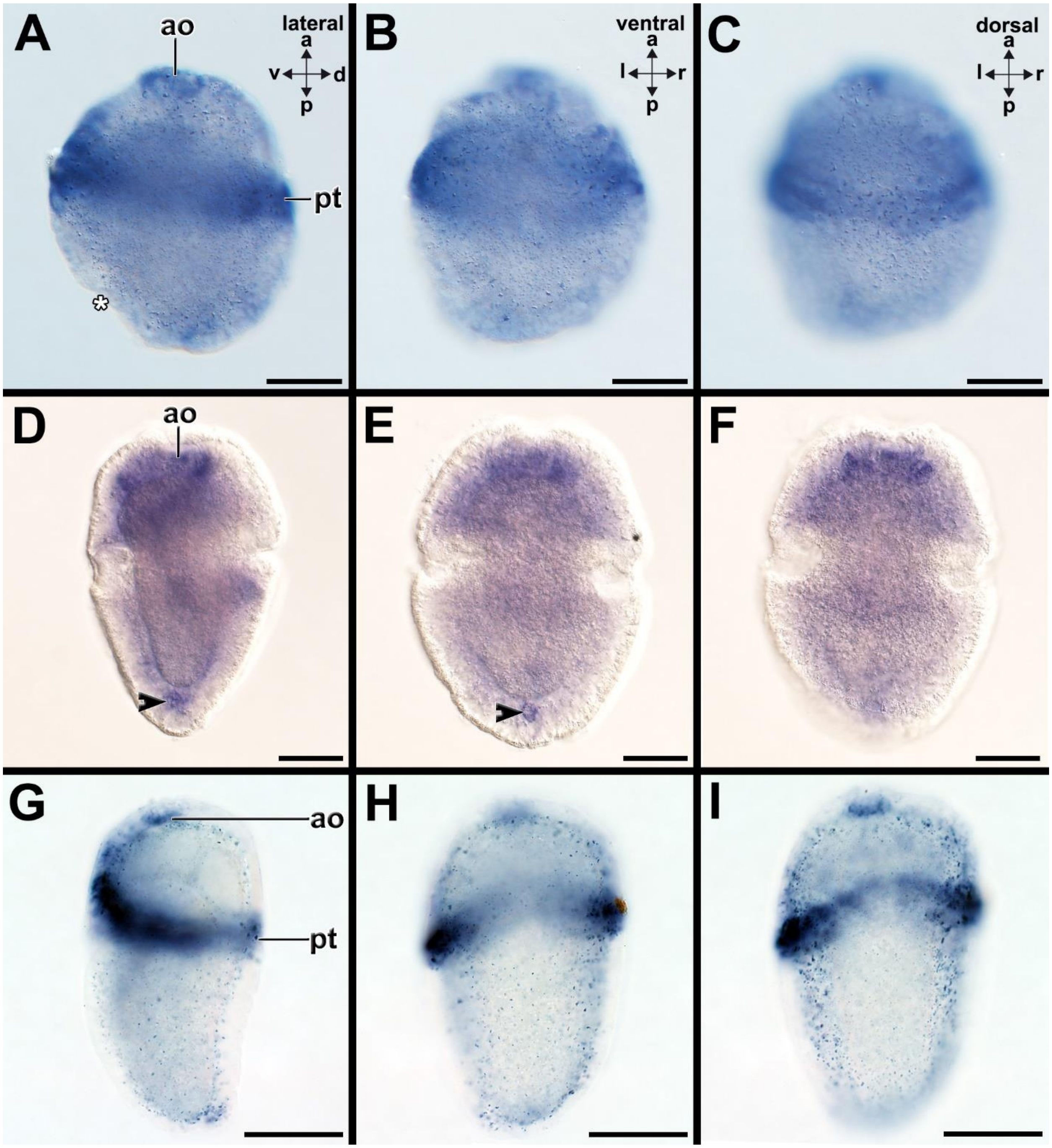

Figure 18 Expression of *ferritin* in the polyplacophoran *Acanthochitona fascicularis.* Dorsal (d)-ventral (v), anterior (a)-posterior (p), and left (l)-right (r) axes indicate the orientation. Asterisk marks the mouth opening. **A-C:** Early trochophore larvae of the polyplacophoran *A. fascicularis* express *fer* in cells of the apical organ (ao) and the prototroch (pt). *Fer* is also faintly expressed in the entire embryo. **D-F:** Mid-stage trochophores express *fer* in cells of the apical organ and few cells in the posterior-most region

(arrowheads). **G-I:** Late-stage trochophores express *fer* in the cells of the prototroch and the apical organ.

Scale bars: 50 µm

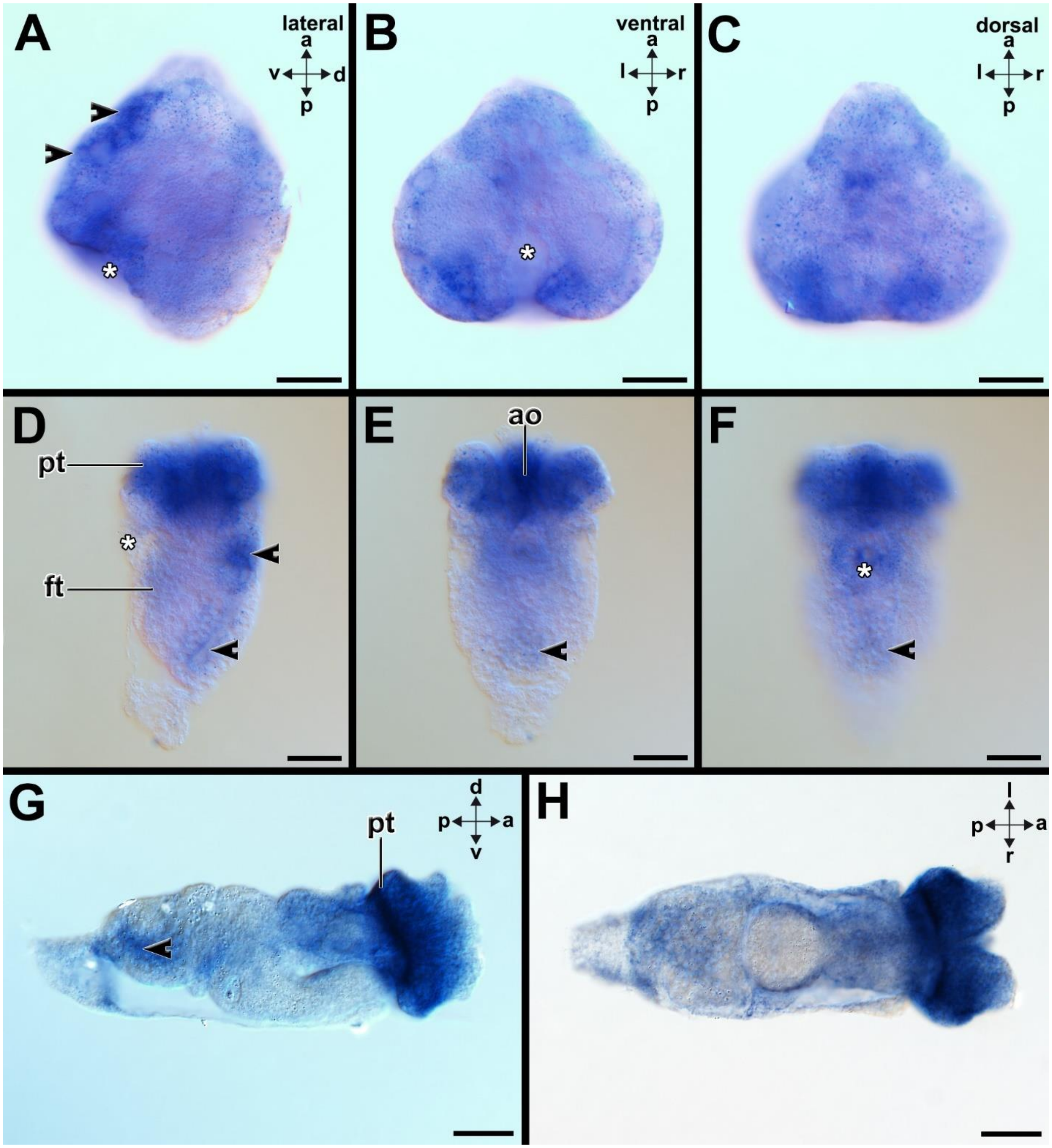

Figure 19 Expression of *ferritin* in the scaphopod *Antalis entalis.* Dorsal (d)-ventral (v), anterior (a)-posterior (p), and left (l)-right (r) axes indicate the orientation. Asterisks mark the mouth opening and foregut in F. **A-C:** *Fer* is expressed around the mouth in early-stage trochophores. Additional *fer*+ cells are distributed along the ventral episphere (arrowheads) and globally throughout the embryo. **D-F:** In mid-stage trochophores *fer* is expressed in the apical organ (ao), the prototroch (pt) and the glandular cells

of the mid- and hindgut (arrowheads). **G-H:** Late-stage trochophores express *fer* in the prototroch and the glandular tissue of the mid- hindgut (arrowheads). Scale bars: 50 μm

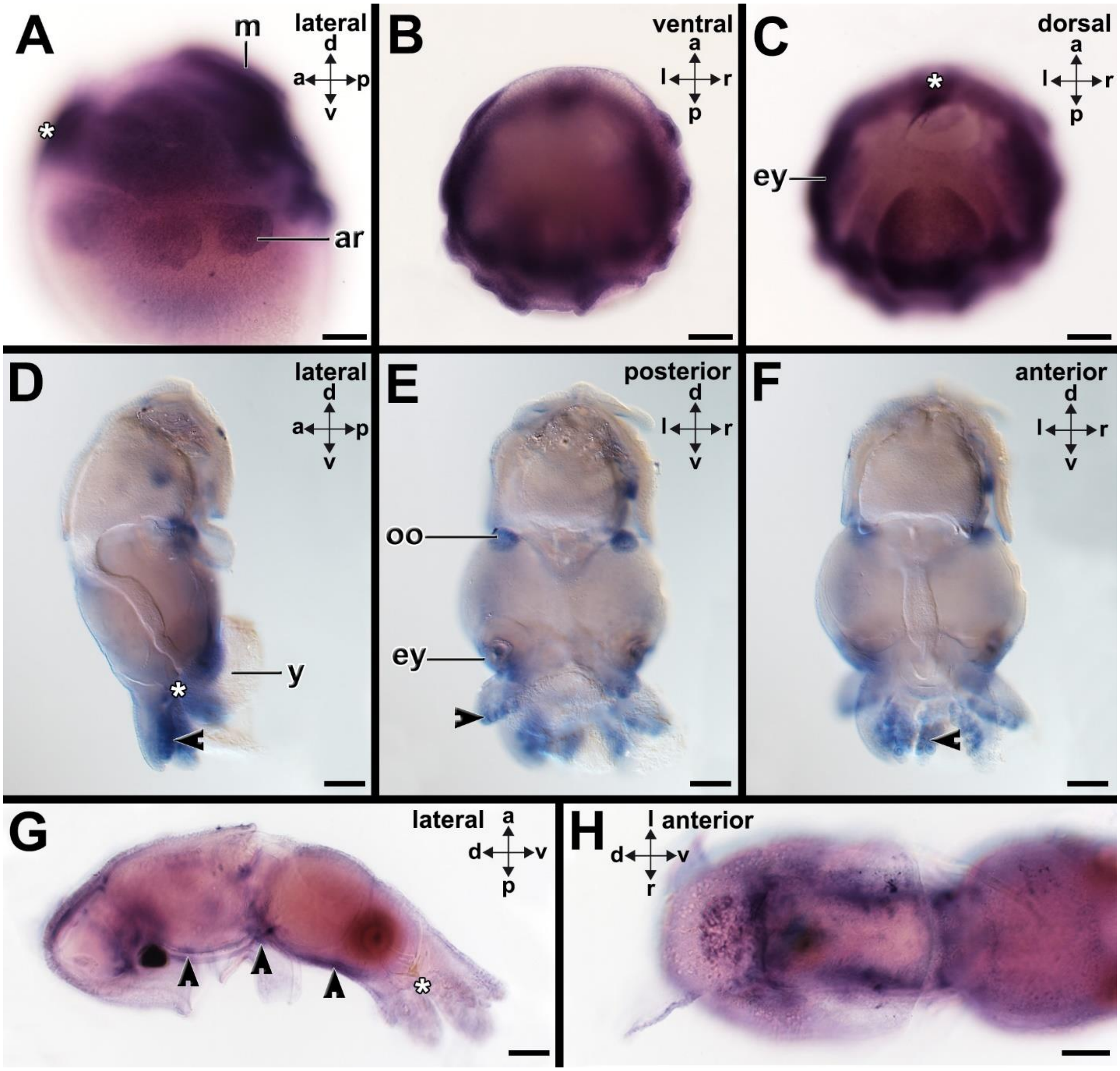

Figure 20 Expression of *ferritin* in the cephalopod *Xipholeptos notoides.* Dorsal (d)-ventral (v), anterior (a)-posterior (p), and left (l)-right (r) axes indicate the orientation. Asterisks mark the mouth opening. **A-C:** In the cephalopod *X. notoides*, early developmental stage 19 expresses *fer* globally in the epidermal layers of various organ systems such as the mantle (m), the arms (ar), the eyes (ey), or the gills. **D-F:** The more advanced developmental stage 25 individuals express *fer* in the suckers (arrowheads), and the olfactory organ (oo). **G-I:** Stage 28 individuals express *fer* in the posterior regions (arrowheads) of the animals. Abbreviation: y, yolk. Scale bars: 100 µm

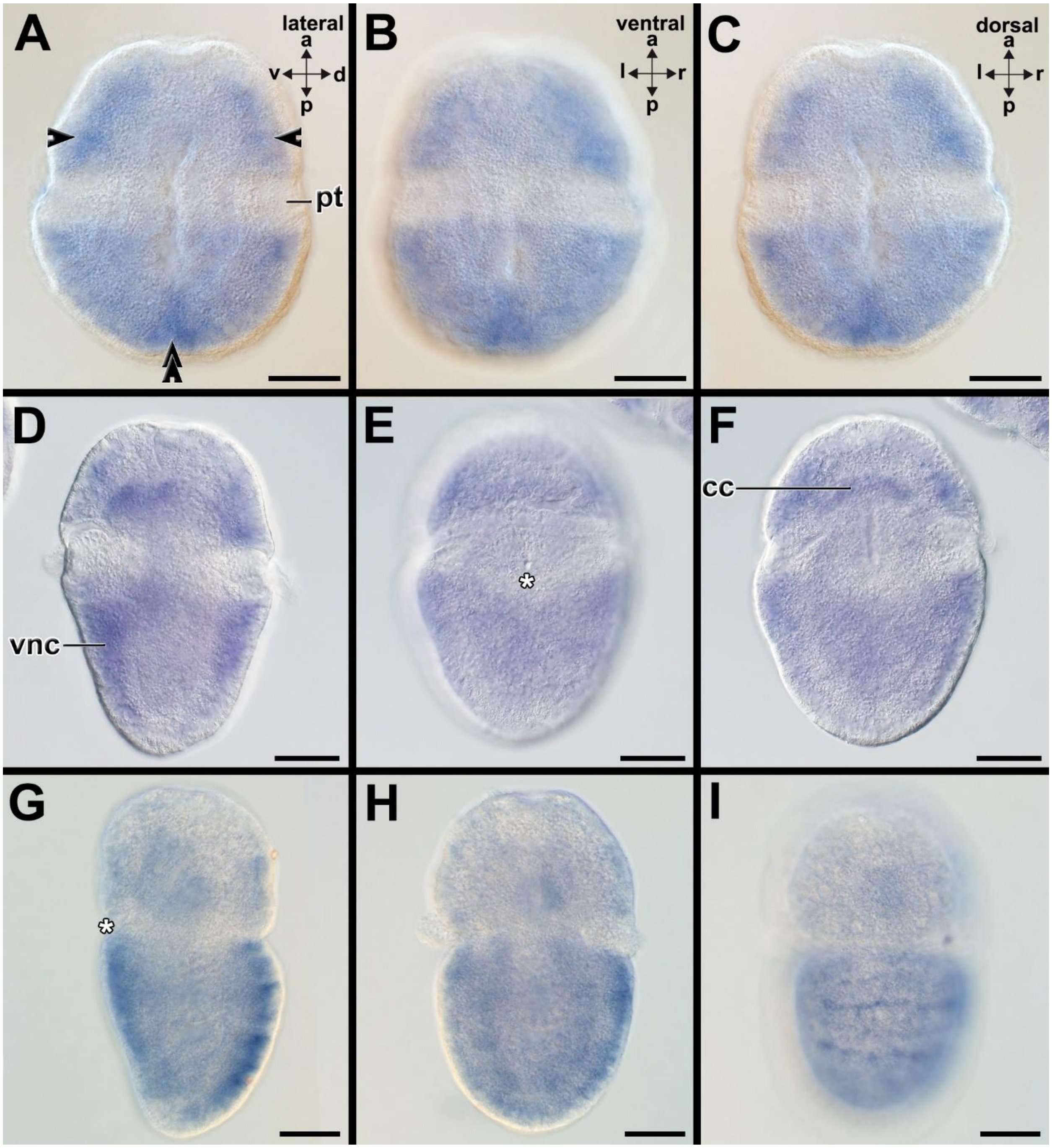

Figure 21 Expression of *notch* in the polyplacophoran *Acanthochitona fascicularis.* Dorsal (d)-ventral (v), anterior (a)-posterior (p), and left (l)-right (r) axes indicate the orientation. Asterisks mark the mouth opening. **A-C:** In early trochophores, *notch* is expressed in the ectodermal layer of the episphere (arrowheads), in the posterior-most medium hyposphere (double-arrowhead), and adjacent to the prototroch (pt) in the hyposphere. **D-F:** In mid-stage trochophore larvae, *notch*-expression is found in the

region of the forming ventral nerve cords (vnc) and the shell fields. In addition, *notch*-expression is present posterior to the apical organ, i.e. the region of the forming cerebral commissure (cc). **G-I:** In late-stage trochophore larvae *notch*-expression is restricted to the ventral nerve cords, the shell fields, and spicules-bearing cells. Scale bars: 50 μm

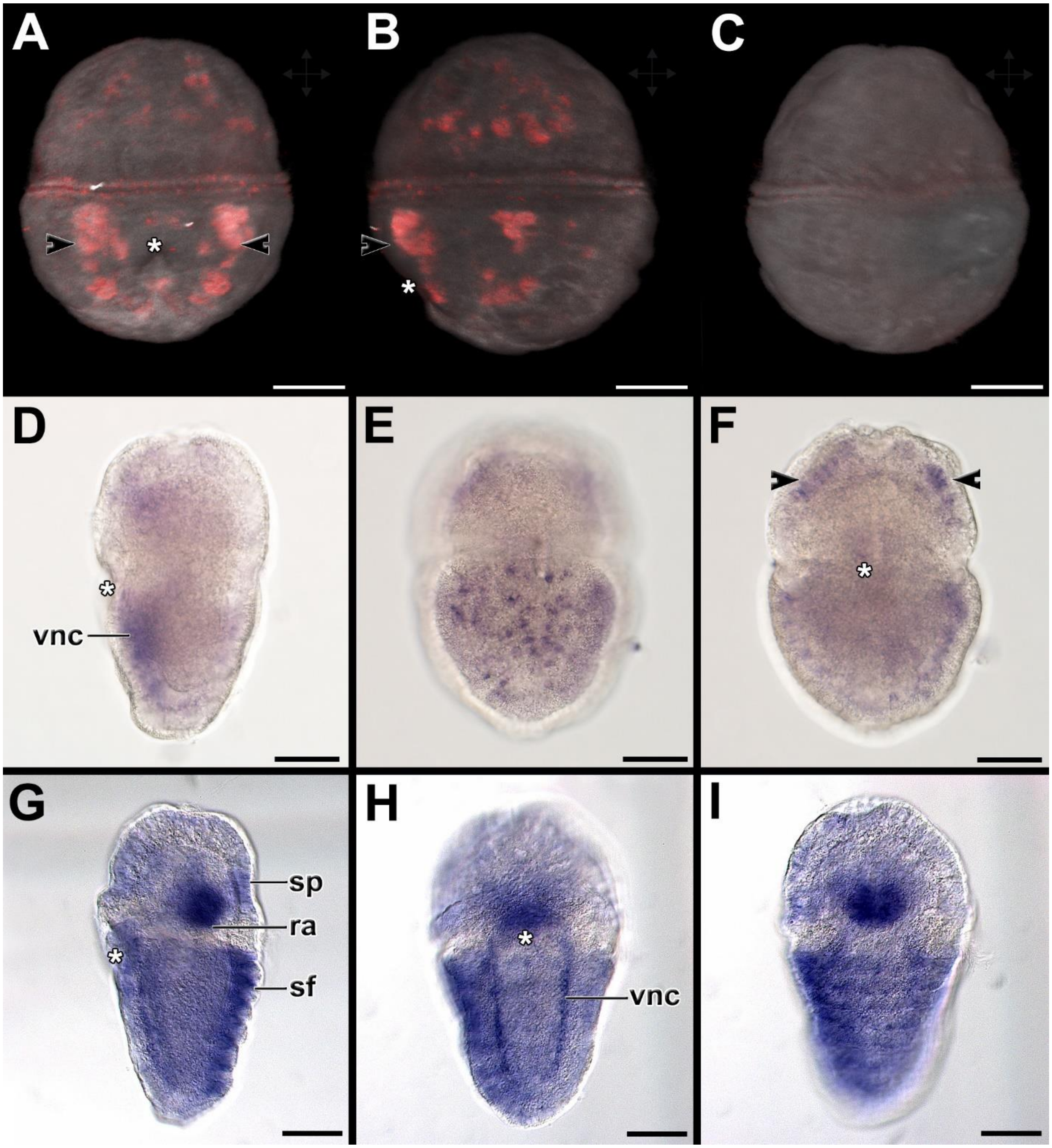

Figure 22 Expression of *delta* in the polyplacophoran *Acanthochitona fascicularis.* Dorsal (d)-ventral (v), anterior (a)-posterior (p), and left (l)-right (r) axes indicate the orientation. Asterisks mark the mouth opening. **A-C:** Early-stage trochophore larvae express *delta* in two regions flanking the mouth opening (arrowheads). Other *delta+* cells are present in ectodermal domains of the episphere. The dorsal side of the early trochophore larvae is devoid of *delta+* cells. Confocal reflection scans with delta+ cells in red

and cell nuclei (DAPI in grey). **D-F:** Mid-stage trochophore larvae express *delta* in the region of the forming ventral nerve cords (vnc) in the ventral hyposphere and in ectodermal cells of the median lateral episphere (arrowheads). **G-I:** *Delta+* cells are present in the shell fields (sf), the spicule-bearing cells (sp), the ventral nerve cords, and in the region of the anlage of the radula (ra). Scale bars: 50 µm

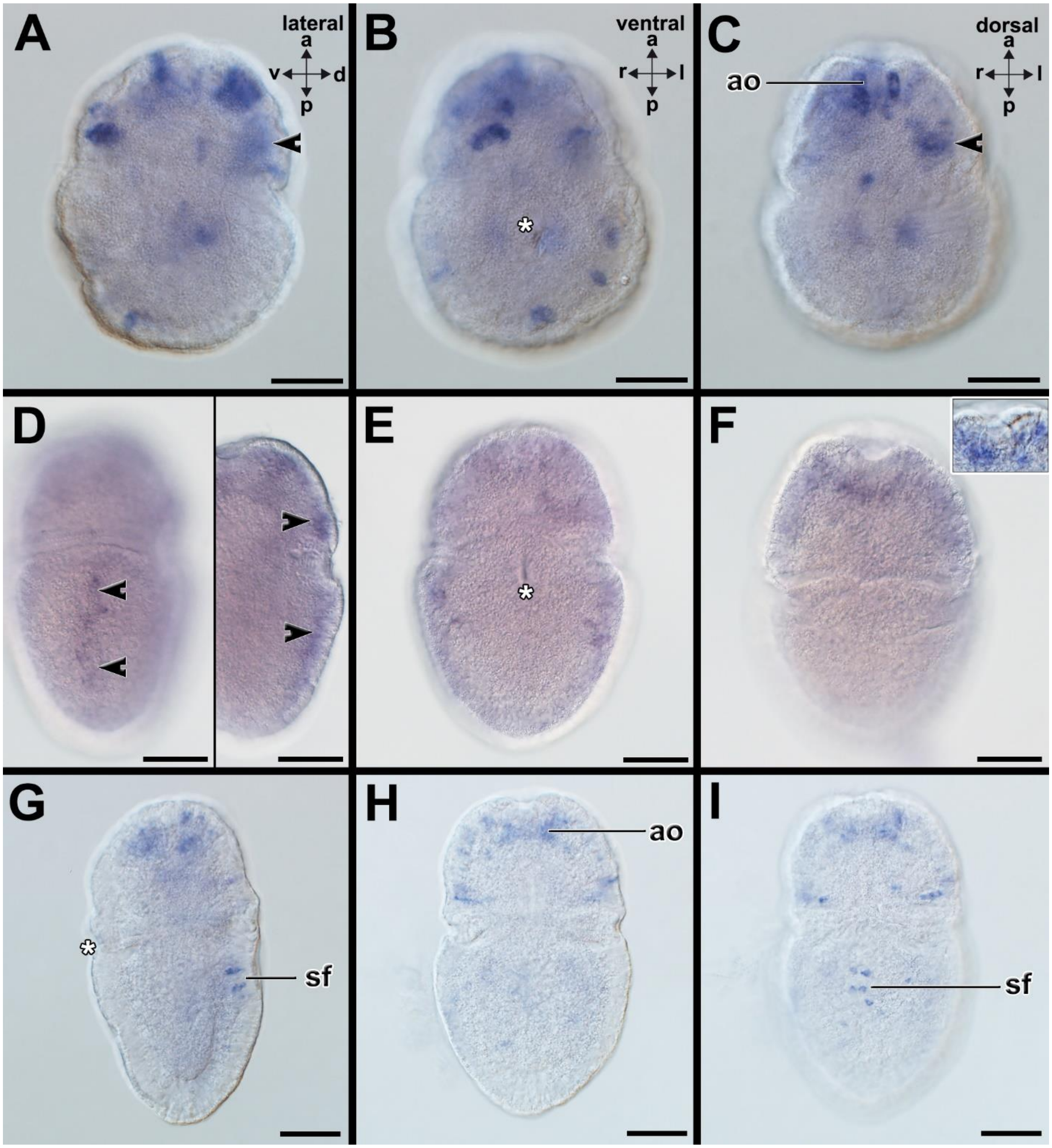

Figure 23 Expression of *zic* in the polyplacophoran *Acanthochitona fascicularis.* Dorsal (d)-ventral (v), anterior (a)-posterior (p), and left (l)-right (r) axes indicate the orientation. Asterisks mark the mouth opening. **A-C:** Early trochophore express *zic* in the episphere, among others in the apical organ (ao). Few cells are present in the hyposphere in the region of the nascent foot but not in the region of the future shell fields. Some *zic*+ cells are located in the region that may give rise to the spicules (arrowheads). **D-F:**

Mid-stage trochophore larvae express *zic* in their spicule-bearing cells (arrowheads) of the perinotum of the lateral hyposphere and in few spicule bearing cells of the episphere (not shown). In addition, *zic* is expressed in the apical organ (inset in F), in the ventral side posterior to the mouth in the foot (right inset in D). **G-I:** Late-stage trochophore larvae express *zic* in the region around the apical organ, in few cells anterior to the prototroch which may correspond to spicule-bearing cells (arrowheads) and in few cells of the shell fields (sf). Scale bars: 50 μm

Table 1. Accession numbers for the sequences used in the orthology analysis. The taxonomic names are, in some cases, followed by the number used in the abbreviation in the orthology analysis.

**Gene**	**Organism**	**Identifiers**
Zic	*Acanthochitona fascicularis*	PQ360980
	Amphioctopus fangsiao	AB231875
	Capitella teleta	HM235720
	Dicyema acuticephalum	AB266039
	Halocynthia roretzi	BAC23063
	Heterololigo bleekeri	AB231874
	Monodelphis domestica	XP_001376758
	Nematostella vectensis	AB231867
	Pandinus imperator	AB231877
	Saccoglossus kowalevskii	NP_001158430
	Strongylocentrotus purpuratus	XP_792929
	Trichoplax adhaerens	XP_002108473
Delta	*Acanthochitona fascicularis*	PQ360978
	Branchiostoma floridae	XP_002590071.1

	Drosophila melanogaster	NP_001247193.1
	Gryllus bimaculatus	BAK53861.1
	Mytilus galloprovincialis	VDI73605.1
	Octopus bimaculoides	XP_014780734.2
	Nematostella vectensis	AEW42992.1
	Penaeus vannamei	AYC35171.1
	Schistosoma mansoni	XP_018655106.1
Goosecoid	*Acanthochitona fascicularis*	PQ360983
	Antalis entalis	PQ360986
	Branchiostoma floridae	AF281674
	Drosophila melanogaster	S70617
	Danio rerio	P53544
	Gallus gallus	A47539
	Gallus gallus	CAA70980.1
	Hylaeus anthracinus	XP_053995374.1
	Holothuria leucospilota	KAJ8044578.1
	Homo sapiens	AAC39544

	Hydra vulgaris	AF183398
	Mus musculus	A42768
	Mus musculus	P56916
	Ptychodera flava	QBZ28539.1
	Patella vulgata	XP_050405189.1
	Trichoplax sp.	RDD44642.1
	Wirenia argentea 1	PQ360991
	Wirenia argentea 2	PQ360990
	Wirenia argentea 3	PQ360992
	Xenopus laevis	B42768
Ferritin	*Acanthochitona fascicularis*	PQ360981
	Antalis entalis	PQ360984
	Aquarana catesbeiana	ACO52052.1
	Aquarana catesbeiana	ACO51930.1
	Aquarana catesbeiana	AAA49525.1
	Andrias davidianus	ALE30608.1
	Andrias davidianus	ALE30610.1

	Andrias davidianus	ALE30609.1
	Branchiostoma floridae	XP_035685992.1
	Caenorhabditis elegans	NP_491198.1
	Callorhinchus milii	NP_001279768.1
	Callorhinchus milii	XP_007883738.1
	Chlorocebus sabaeus	XP_007989558.1
	Ciona savignyi	H2YP89
	Eptatretus burgeri	ENSEBUP00000001471
	Eptatretus burgeri	ENSEBUP00000024901
	Homo sapiens	NP_803431.1
	Haemaphysalis longicornis	AB734098.1
	Homo sapiens	NP_002023.2
	Homo sapiens	NP_000137.2
	Homo sapiens	NP_114100.1
	Ixodes ricinus	ACJ70653.1
	Latimeria chalumnae	H3BD48
	Latimeria chalumnae	H3BE79

	Latimeria chalumnae	H3A323
	Lepidosiren paradoxa	GEHZ01029327.1
	Lepidosiren paradoxa	GEHZ01038500.1
	Lepidosiren paradoxa	GEHZ01089515.1
	Mus musculus	NP_034369.1
	Mus musculus	NP_034370.2
	Mus musculus	NP_112551.2
	Mus musculus	NP_080562.2
	Oikopleura dioica	CBY35180.1
	Oryzias latipes	XP_020569048.1
	Oryzias latipes	XP_004071907.1
	Petromyzon marinus	XP_032813987.1
	Petromyzon marinus	XP_032813876.1
	Petromyzon marinus	XP_032813875.1
	Petromyzon marinus	XP_032830481.1
	Rhincodon typus	XP_020383843.1
	Rhincodon typus	XP_020392390.1

	Squalus acanthias	CX196387.1
	Squalus acanthias	ES651741.1
	Strongylocentrotus purpuratus	XP_030844960.1
	Xenopus tropicalis	NP_001005135.1
	Xenopus tropicalis	NP_989212.1
	Xenopus tropicalis	NP_989008.1
	Xipholeptos notoides	PQ360987
Chitin- synthase	*Acanthochitona fascicularis*	PQ360976
	Aspergillus fumigatus	sequence obtained from Zakrzewski et al., 2014
	Anopheles gambiae	sequence obtained from Zakrzewski et al., 2014
	Amphimedon queenslandica	sequence obtained from Zakrzewski et al., 2014
	Atrina rigida	sequence obtained from Zakrzewski et al., 2014
	Branchiostoma floridae	sequence obtained from Zakrzewski et al., 2014

	Brugia malayi	sequence obtained from Zakrzewski et al., 2014
	Bos taurus	sequence obtained from Zakrzewski et al., 2014
	Caenorhabditis elegans	sequence obtained from Zakrzewski et al., 2014
	Ciona intestinalis	sequence obtained from Zakrzewski et al., 2014
	Capitella teleta	sequence obtained from Zakrzewski et al., 2014
	Dirofilaria immitis	sequence obtained from Zakrzewski et al., 2014
	Drosophila melanogaster	sequence obtained from Zakrzewski et al., 2014
	Danio rerio	sequence obtained from Zakrzewski et al., 2014
	Hydra magnipapillata	sequence obtained from Zakrzewski et al., 2014
	Leptochiton asellus	sequence obtained from Zakrzewski et al., 2014

	Leucosolenia complicata	sequence obtained from Zakrzewski et al., 2014
	Lottia gigantea	sequence obtained from Zakrzewski et al., 2014
	Meloidogyne artiellia	sequence obtained from Zakrzewski et al., 2014
	Monosiga brevicollis	sequence obtained from Zakrzewski et al., 2014
	Myzostoma cirriferum	sequence obtained from Zakrzewski et al., 2014
	Macandrevia cranium	sequence obtained from Zakrzewski et al., 2014
	Mytilus galloprovincialis]	sequence obtained from Zakrzewski et al., 2014
	Manduca sexta	sequence obtained from Zakrzewski et al., 2014
	Neurospora crassa	sequence obtained from Zakrzewski et al., 2014
	Nematostella vectensis	sequence obtained from Zakrzewski et al., 2014

	Owenia fusiformis	sequence obtained from Zakrzewski et al., 2014
	Ostrinia furnacalis	sequence obtained from Zakrzewski et al., 2014
	Platynereis dumerili	sequence obtained from Zakrzewski et al., 2014
	Pinctada fucata	sequence obtained from Zakrzewski et al., 2014
	Sabellaria alveolata	sequence obtained from Zakrzewski et al., 2014
	Sycon ciliatum	sequence obtained from Zakrzewski et al., 2014
	Salpingoeca sp.	sequence obtained from Zakrzewski et al., 2014
	Tribolium castaneum	sequence obtained from Zakrzewski et al., 2014
	Trichinella spiralis	sequence obtained from Zakrzewski et al., 2014
	Wirenia argentea	PQ360977

	Xenopus tropicalis	sequence obtained from Zakrzewski et al., 2014
Notch	*Aedes aegypti*	sequence obtained from Gazave et al., 2009
	Acanthochitona fascicularis	PQ360979
	Amphimedon queenslandica	sequence obtained from Gazave et al., 2009
	Branchiostoma floridae	sequence obtained from Gazave et al., 2009
	Caenorhabditis elegans	sequence obtained from Gazave et al., 2009
	Ciona intestinalis	sequence obtained from Gazave et al., 2009
	Danio rerio	sequence obtained from Gazave et al., 2009
	Gallus gallus	sequence obtained from Gazave et al., 2009
	Halocynthia roretzi	sequence obtained from Gazave et al., 2009

	Homo sapiens	sequence obtained from Gazave et al., 2009
	Lottia gigantea	sequence obtained from Gazave et al., 2009
	Nematostella vectensis	sequence obtained from Gazave et al., 2009
	Strongylocentrotus purpuratus	sequence obtained from Gazave et al., 2009
	Trichoplax adhaerens	sequence obtained from Gazave et al., 2009
	Xenopus tropicalis	sequence obtained from Gazave et al., 2009
Otx	*Branchiostoma floridae*	AAC00193.1
Zap	*Nakaseomyces glabratus*	XP_447926
	Saccharomyces cerevisiae	NP_012479
Bacterioferritin	*Cyanobacterium aponium*	AFZ54996.1
	Thiothrix nivea	EIJ33617.1
Jagged	*Ciona intestinalis*	XP_009860298.2
	Oikoplura dioica	CBY19564.1

	Mytilus galloprovincialis	VDI74143.1
Grainyhead	*Acanthochitona fascicularis*	PQ360982
	Anopheles gambiae	XP_308698.4
	Antalis entalis	PQ360985
	Capitella capitata	jgi| Capca1| 198092| fgenesh1_pg.C_scaffold_3000030
	Ciona intestinlis	jgi| Cioin2| 262638| gw1.01q.820.1
	Daphnia pulex	jgi| Dappu1| 192185| estExt_Genewise1Plus.C_50175
	Drosophila melanogaster	NP_476842.2
	Homo sapiens	NP_005644.2
	Homo sapiens	AAH67519.1
	Lottia gigantea	jgi| Lotgi1| 157385| fgenesh2_pg.C_sca_14000008
	Nematostella vectensis	jgi| Nemve1| 95157| e_gw.38.94.1
	Nipponacmea fuscoviridis	BAK40160.1
	Phycomyces blakesleeanus	jgi| Phybl1| 75515| estExt_fgeneshPB_pg.C_10328
	Phycomyces blakesleeanus	jgi| Phybl1| 63864| fgeneshPB_pg.8__95
	Trichoplax adhaerens	jgi| Triad1| 25702| e_gw1.5.1214.1
	Wirenia argentea	PQ360989

	Xipholeptos notoides	PQ360988

Table 2. Abbreviations of the species names used in the orthology analysis tree.

Species name	***Abbreviation***
Acanthochitona fascicularis	*Acfa*
Aedes aegypti	*Aae*
Amphimedon queenslandica	*Aqu*
Amphioctopus fangsiao	*Afa*
Andrias davidianus	*Ada*
Anopheles gambiae	*Aga*
Antalis entalis	*Aen*
Aquarana catesbeiana	*Aca*
Aspergillus fumigatus	*Afu*
Atrina rigida	*Ari*
Bos taurus	*Bta*
Branchiostoma floridae	*Bfl*
Brugia malayi	*Bma*
Caenorhabditis elegans	*Cel*
Callorhinchus milii	*Cmi*

Capitella capitata	*Cca*
Capitella teleta	*Cte*
Chlorocebus sabaeus	*Chsa*
Ciona intestinalis	*Cin*
Ciona savignyi	*Csa*
Cyanobacterium aponium	*Cap*
Danio rerio	*Dre*
Daphnia pulex	*Dpu*
Dicyema acuticephalum	*Dac*
Dirofilaria immitis	*Dim*
Drosophila melanogaster	*Dme*
Eptatretus burgeri	*Ebu*
Gallus gallus	*Gga*
Gryllus bimaculatus	*Gbi*
Haemaphysalis longicornis	*Hlo*
Halocynthia roretzi	*Hro*
Heterololigo bleekeri	*Hbl*

Holothuria leucospilota	*Hle*
Homo sapiens	*Hsa*
Hydra magnipapillata	*Hma*
Hydra vulgaris	*Hvu*
Hylaeus anthracinus	*Han*
Ixodes ricinus	*Iri*
Latimeria chalumnae	*Lch*
Lepidosiren paradoxa	*Lpa*
Leptochiton asellus	*Las*
Leucosolenia complicata	*Lco*
Lottia gigantea	*Lgi*
Macandrevia cranium	*Mcr*
Manduca sexta	*Mse*
Meloidogyne artiellia	*Mar*
Monodelphis domestica	*Mdo*
Monosiga brevicollis	*Mbr*
Mus musculus	*Mmus*

Mytilus galloprovincialis	*Mga*
Myzostoma cirriferum	*Mci*
Nakaseomyces glabratus	*Ngl*
Nematostella vectensis	*Nve*
Neurospora crassa	*Ncr*
Nipponacmea fuscoviridis	*Nfu*
Octopus bimaculoides	*Obi*
Oikopleura dioica	*Odi*
Oryzias latipes	*Ola*
Ostrinia furnacalis	*Osfu*
Owenia fusiformis	*Ofu*
Pandinus imperator	*Pim*
Paracentrotus lividus	*Pli*
Patella vulgata	*Pvu*
Penaeus vannamei	*Pva*
Petromyzon marinus	*Pma*
Phycomyces blakesleeanus	*Pbl*

Pinctada fucata	*Pfu*
Platynereis dumerili	*Pdu*
Ptychodera flava	*Pga*
Rhincodon typus	*Rty*
Sabellaria alveolata	*Sal*
Saccharomyces cerevisiae	*Sce*
Saccoglossus kowalevskii	*Sko*
Salpingoeca sp.	*Ssp*
Schistosoma mansoni	*Sma*
Squalus acanthias	*Sac*
Strongylocentrotus purpuratus	*Spu*
Sycon ciliatum	*Sci*
Thiothrix nivea	*Tni*
Tribolium castaneum	*Tca*
Trichinella spiralis	*Tsp*
Trichoplax adhaerens	*Tad*
Trichoplax sp.	*Tri*

Wirenia argentea	*War*
Xenopus laevis	*Xla*
Xenopus tropicalis	*Xtr*
Xipholeptos notoides	*Xno*

Table 3. Estimated amino acid substitution models for the analyzed genes.

**Gene**	**Model and rate variation**
notch	Whelan and Goldman substitution model, with gamma-distributed rate variation (WAG+G)
chitin-synthase	Le and Gascuel substitution model, with invariant sites and gamma-distributed rate variation (LG+I+G)
zic	Jones substitution model with gamma-distributed rates (JTT+G)
ferritin	Le and Gascuel substitution model with gamma-distributed rates (LG+G)
goosecoid	Jones substitution model with invariant sites and gamma-distributed rates (JTT+I+G)

grainyhead	Muller and Vingron substitution model, with invariant sites and gamma distributed rates (VT+I+G)

Table 4. Gene expression as revealed by *in situ* hybridization experiments in shell fields, spicule-bearing cells, and chaetoblasts of mollusks, annelids, and brachiopods, respectively. Due to spatial restrictions only few selected references per gene are stated. Abbreviations: A, adult; D, developmental stage; n/a, no expression patterns published to our best knowledge.

**gene/ taxon**	**Mollusca**			
	**Conchifera**			
	**Gastropoda**	**Bivalvia**	**Cephalopoda**	**Scaphopoda**
**Hox genes**				
lab (hox1)	D: surrounding shell field (Hinman et al. 2003; Samadi and Steiner 2009; Huan et al. 2020)	D: surrounding shell fields (Salamanca-Díaz et al. 2021)	D: shell sac (Barrera Grijalba et al., 2022)	D: surrounding shell field (Wollesen et al., 2018)
pb (hox2)	D: shell field (Huan et al., 2020)			D: surrounding shell field (Wollesen et al., 2018)
zen (hox3)	D: shell field (Huan et al., 2020)			D: surrounding shell field (Wollesen et al., 2018)
dfd (hox4)	D: surrounding shell field (Samadi and Steiner 2009; Huan et al. 2020)			D: surrounding shell field (Wollesen et al., 2018)
scr (hox5)	D: shell field (Huan et al., 2020)		D: shell sac (Barrera Grijalba et al., 2022)	
lox5	D: shell field (Huan et al., 2020)			
antp (hox7)				
lox4	D: shell field (Huan et al., 2020)			
lox2	D: shell field (Huan et al., 2020)			
post1	D: surrounding shell field (Samadi and Steiner 2009)			D: surrounding shell field (Wollesen et al., 2018)
post2	D: surrounding shell field (Hinman et al. 2003; Samadi and Steiner 2009; Huan et al. 2020)	D: shell fields (Salamanca-Díaz et al. 2021)		
**Other genes**				
goosecoid	D: mantle margin (Lartillot et al., 2002)		n/a	D: mantle margin (present study)
chitin-synthase			n/a	n/a
grainyhead			D: shell sac (present study)	D: mantle margin (present study)
pax2/5/8	D: anterior mantle (O` Brien & Degnan, 2003)	D: shell fields (Wollesen et al., 2015)	D: shell sac/ entire mantle (Wollesen et al. 2015)	n/a
gbx		D: shell fields (Wollesen et al., 2017)	n/a	n/a
engrailed	D: shell field (Kin et al., 2009; Huan et al., 2020)	D: shell fields (Kin et al., 2009)		
perlucin				
dlx				
sp1				
sp2				
ferritin	D: in mantle (Jackson et al., 2006)		D: in multiple domains, also mantle (present study)	D: in multiple domains, but not mantle (present study)
dpp (bmp2/4)	D: shell field (Nederbragt et al., 2002; Kin et al., 2009)	D: shell fields (Kin et al., 2009)	n/a	n/a
notch				
delta				
zic				
hes				
arx				

**gene/ taxon**			**Annelida**	**Brachiopoda**
	**Aculifera**			
	**Polyplacophora**	**Neomeniomorpha**		
**Hox genes**				
lab (hox1)	D: 1st shell field (Fritsch et al., 2015, 2016; Huan et al. 2020)	D: spicule-bearing cells (present study)		D: chaetal sacs (Schiemann et al., 2017)
pb (hox2)	D: shell fields (Fritsch et al., 2015, 2016; Huan et al., 2020)			
zen (hox3)	D: shell fields (Fritsch et al., 2015, 2016; Huan et al., 2020)			
dfd (hox4)	D: shell fields (Fritsch et al., 2015, 2016; Huan et al., 2020)			
scr (hox5)	D: shell fields (Huan et al., 2020)			D: shell-forming epithelium (periostracum) (Schiemann et al., 2017)
lox5	D: shell fields (Fritsch et al., 2015, 2016; Huan et al., 2020)			
antp (hox7)	D: shell fields (Fritsch et al., 2015, 2016; Huan et al., 2020)			D: shell-forming epithelium (periostracum) (Schiemann et al., 2017)
lox4	D: shell fields (Fritsch et al., 2015, 2016; Huan et al., 2020)			
lox2	D: shell fields (Fritsch et al., 2015, 2016; Huan et al., 2020)			
post1				D: chaetal sacs (Schiemann et al., 2017)
post2	D: shell fields (Fritsch et al., 2015, 2016; Huan et al., 2020)			
**Other genes**				
goosecoid	D: spicule-bearing cells (present study)	D: spicule-bearing cells (present study)		
chitin-synthase	present study	present study		
grainyhead	D: spicule-bearing cells (present study)	D: spicule-bearing cells (present study)		
pax2/5/8	D: shell fields and spicule-bearing cells (Wollesen et al., 2015)	n/a		
gbx	D: shell fields and spicule-bearing cells (Wollesen et al., 2017)	D: spicule-bearing cells (Wollesen et al., 2017)		
engrailed	D: shell fields and spicule-bearing cells (Huan et al., 2020)			mantle margin (Wernström et al., 2022)
perlucin				mantle margin (Wernström et al., 2022)
dlx				mantle margin (Wernström et al., 2022)
sp1				mantle margin (Wernström et al., 2022)
sp2				mantle margin (Wernström et al., 2022)
ferritin	D: in multiple domains, but not mantle (present study)			entire larva, also mantle (Wernström et al., 2022)
dpp (bmp2/4)	n/a	n/a		not in mantle (Wernström et al. 2022) n/a but see psmad-immunoreactivity in shell fields (Luo et al. 2015)
notch	present study	n/a	D: chaetae (Gazave et al., 2009; Schiemann et al., 2017)	D: chaetae (Schiemann et al., 2017)
delta	present study	n/a	D: chaetae (Schiemann et al., 2017)	D: chaetae (Schiemann et al., 2017)
zic	present study	n/a	D: chaetae (Schiemann et al., 2017)	D: chaetae (Schiemann et al., 2017)
hes	?	n/a	D: chaetae (Schiemann et al., 2017)	D: chaetae (Schiemann et al., 2017)
arx		n/a	D: chaetae (Schiemann et al., 2017)	D: chaetae (Schiemann et al., 2017)

References cited in the Supplementary Information:

Barrera Grijalba, C. C., Rodríguez Monje, S. V., Gestal, C., & Wollesen, T. Octopod Hox genes and cephalopod plesiomorphies. *Scientific Reports*, 13, 15492 (2023).

Hinman, V. F., O´Brien, E. K., Richards, G. S., Degnan, B. M. Expression of anterior Hox genes during larval development of the gastropod *Haliotis asinina*. *Evolution & Development*, 5, 5 508-521 (2003).

Huan, P., Wang, Q., Tan, S., & Liu, B. Dorsoventral decoupling of Hox gene expression underpins the diversification of molluscs. *PNAS*, 117(1), 503-512 (2020).

Gazave, E., Lapébie, P., Richards, G. S., Brunet, F., Ereskovsky, A. V., Degnan, B. M., Borchiellini, C., Vervoort, M., Renard, E. Origin and evolution of the Notch signalling pathway: an overview from eukaryotic genomes. *BMC Evolutionary Biology* 13, 9, 249 (2009).

Jackson, D. J., McDougall, C., Green, K., Simpson, F., Wörheide, G., Degnan, B. M. A rapidly evolving secretome builds and patterns a sea shell. *BMC Biology*, 4, 40 (2006).

Kin, K., Kakoi, S., Wada, H. A novel role for *dpp* in the shaping of bivalve shells revealed in a conserved molluscan developmental program. *Developmental biology* 329, 152-166.

Lartillot, N., Le Gouar, M., Adoutte, A. Expression of *fork head* and *goosecoid* homologues in the mollusc *Patella vulgata* supports the ancestry of the anterior mesendoderm across Bilateria. *Development Genes and Evolution* 212, 551-561 (2002).

Luo, Y. J., et al. The *Lingula* genome provides insights into brachiopod evolution and the origin of phosphate biomineralization. *Nature communications* 6, 8301 (2015).

Nederbragt, A. J., van Loon, A. E., Dictus, W. J. A. G. Expression of *Patella vulgata* orthologs of *engrailed* and *dpp-BMP2/4* in adjacent domains during molluscan shell development suggests a conserved compartment boundary mechanism. *Developmental Biology* 246, 341-355 (2002).

O`Brien, E. K., Degnan, B. M. Expression of *Pax2/5/8* in the gastropod statocyst: insights into the antiquity of metazoan geosensory organs. *Evolution & Development* 5, 6, 572-578 (2003)

Samadi, L., & Steiner, G. Involvement of Hox genes in shell morphogenesis in the encapsulated development of a top shell gastropod (*Gibbula varia* L.). *Development Genes and Evolution*, 219, 523-530 (2009).

Salamanca-Díaz, D. A., Calcino, A. D., de Oliveira, A. L., & Wanninger, A. Non-collinear Hox gene expression in bivalves and the evolution of morphological novelties in mollusks. *Scientific Reports*, 11, 3575 (2021).

Schiemann, S. M., Martín-Durán, J. M., Børve, A., Vellutini, B. C., Passamaneck, Y. J., & Hejnol, A. Clustered brachiopod Hox genes are not expressed collinearly and are associated with lophotrochozoan novelties. *PNAS*, 114(10), E1913-E1922 (2017).

Wernström, J. V., Gasiorowski, L., Hejnol, A. Brachiopod and molluscan biomineralization is a conserved process that was lost in the phoronid-bryozoan stem lineage. *EvoDevo*, 13, 17 (2022).

Wollesen, T., Rodríguez Monje, S. V., Todt, C., Degnan, B. M., & Wanninger, A. Ancestral role of Pax2/5/8 in molluscan brain and multimodal sensory system development. *BMC Evolutionary Biology*, 15, 231 (2015).

Wollesen, T., Scherholz, M., Rodríguez Monje, S. V., Redl, E., Todt, C., & Wanninger, A. Brain regionalization genes are coopted into shell field patterning in Mollusca. *Scientific Reports*, 7, 5486 (2017).

Wollesen, T., Rodríguez Monje, S. V., Luiz de Oliveira, A., & Wanninger, A. Staggered Hox expression is more widespread among molluscs than previously appreciated. *Proceedings of the Royal Society B*, 285, 20181513 (2018).

Zakrzewski, A. C., Weigert, A., Helm, C., Adamski, M., Adamska, M., Bleidorn, C., Raible, F., Hausen, H. Early Divergence, Broad Distribution, and High Diversity of Animal Chitin Synthases. *Genome Biology and Evolution* 6, 316-325 (2014).
